# Delineating Ultrafast Structural Dynamics of a Green-Red Fluorescent Protein for Calcium Sensing

**DOI:** 10.3390/bios13020218

**Published:** 2023-02-02

**Authors:** Taylor D. Krueger, Longteng Tang, Chong Fang

**Affiliations:** Department of Chemistry, Oregon State University, 153 Gilbert Hall, Corvallis, OR 97331, USA

**Keywords:** fluorescent protein-based biosensors, calcium sensing, ultrafast molecular spectroscopy, femtosecond stimulated Raman spectroscopy, protein rational design

## Abstract

Fluorescent proteins (FPs) are indispensable tools for noninvasive bioimaging and sensing. Measuring the free cellular calcium (Ca^2+^) concentrations *in vivo* with genetically encodable FPs can be a relatively direct measure of neuronal activity due to the complex signaling role of these ions. REX-GECO1 is a recently developed red-green emission and excitation ratiometric FP-based biosensor that achieves a high dynamic range due to differences in the chromophore response to light excitation with and without calcium ions. Using steady-state electronic measurements (UV/Visible absorption and emission), along with time-resolved spectroscopic techniques including femtosecond transient absorption (fs-TA) and femtosecond stimulated Raman spectroscopy (FSRS), the potential energy surfaces of these unique biosensors are unveiled with vivid details. The ground-state structural characterization of the Ca^2+^-free biosensor via FSRS reveals a more spacious protein pocket that allows the chromophore to efficiently twist and reach a dark state. In contrast, the more compressed cavity within the Ca^2+^-bound biosensor results in a more heterogeneous distribution of chromophore populations that results in multi-step excited state proton transfer (ESPT) pathways on the sub-140 fs, 600 fs, and 3 ps timescales. These results enable rational design strategies to enlarge the spectral separation between the protonated/deprotonated forms and the Stokes shift leading to a larger dynamic range and potentially higher fluorescence quantum yield, which should be broadly applicable to the calcium imaging and biosensor communities.

## 1. Introduction

Calcium ions (Ca^2+^) serve a diverse array of functions in living systems, including cellular signaling and regulating biochemical processes as ubiquitous secondary messengers [1,2,3]. Calcium sensing is of particular interest to the neuroscientific community because measurements of free cellular calcium can be a relatively direct correlation to neuronal activity [4,5,6,7]. Combined with recent innovations in modern imaging techniques, fluorescence microscopy has become an integral toolset to map out the neuronal circuitry *in vivo* [8,9,10,11]. On the indicator side, the continued improvement of genetically encodable biosensors has allowed for noninvasive techniques to image deep tissue in a living organism with (sub)-cellular specificity [12,13,14,15]. There are several desirable properties for genetically encoded calcium indicators (GECIs) that include a large dynamic range and Stokes shift, long emission wavelength, enhanced affinity for calcium ions over other similar ions, and fast response time [10,16,17,18]. Among the calcium biosensors, there are primarily two types of indicators: Förster resonance energy transfer (FRET) between a pair of biosensors, and the GECI called GCaMP (with GFP, calmodulin/CaM, and a myosin light-chain kinase M13 peptide) types [19,20,21,22,23,24,25]. The latter indicator relies on the Ca^2+^-mediated modulation of the fluorescence properties of a single biosensor as the fluorophore [23,24,26,27,28], while the tyrosine-derived FP chromophore is the photosensory core and emitting species. In addition to the aforementioned properties, a ratiometric (either excitation or emission) indicator is preferred to alleviate undesirable experimental artifacts from complex experimental conditions. While FRET-based biosensors are intrinsically ratiometric, they often suffer from relatively small dynamic ranges. Therefore, many GCaMP-type single FP-based ratiometric Ca^2+^ indicators have been engineered with superior dynamic ranges including GEX-GECO1, GEM-GECO1, and Y-GECOs (GECO stands for genetically encodable calcium indicators for optical imaging) [23,29,30,31,32,33].

Following the engineering of a red-shifted Ca^2+^-biosensor, R-GECO1 [20], a unique excitation-ratiometric indicator was designed in recent years: REX-GECO1 that is an excitation-ratiometric red fluorescent protein (RFP)-CaM chimeric protein biosensor capable of achieving a red/green emission ratio change of ~300% in cultured human cervical cancer (HeLa) cells, although there is room for improvement, particularly regarding the fluorescence quantum yield (FQY) of both green and red forms as well as the dynamic range [18,27]. The red-shifted emission of REX-GECO1 with a large Stokes shift and dynamic range makes the biosensor highly advantageous for real-time dynamic Ca^2+^ imaging, with the demonstrated potential to combine with the GFP-based indicators for simultaneous multicolor one- and two-photon imaging [27]. A more recent report revealed that the working mechanism of the Ca^2+^-bound biosensor is intimately related to a nearby residue, Glu80, which alters the excited-state energy dissipation pathways following photoexcitation [18]. In the current investigation, we delve deeper into the electronic and structural characterization of REX-GECO1 with and without calcium ions *in vitro* via steady-state (UV/Visible absorption and fluorescence emission) and time-resolved (femtosecond transient absorption, abbreviated as fs-TA) electronic spectroscopies in tandem with the ground- and excited-state vibrational techniques by implementing the wavelength-tunable femtosecond stimulated Raman spectroscopy (FSRS) [34,35,36,37,38]. Near-UV (e.g., 400 nm) excitation of REX-GECO1 allows for in-depth analysis of the Ca^2+^-free biosensor to further evaluate the potential for an ultrafast excited-state proton transfer (ESPT) event, a lingering question from the prior investigation, in addition to the photoinduced dark state formation on ultrafast timescales. Structural characterization via ground-state FSRS of the Ca^2+^-free and bound REX-GECO1 reveals subtle differences in the chromophore at thermal equilibrium due to different chromophore-local environment interactions. Furthermore, the excited-state FSRS reveals a multi-step ultrafast ESPT pathway when the biosensor is bound to calcium ions, while certain characteristic chromophore twisting motions lead to efficient dark state formation of the Ca^2+^-free biosensor on the few picosecond (ps) timescale in neutral aqueous buffer. With these novel spectroscopic insights at hand, we develop several rational design strategies to facilitate protein engineers to improve the dynamic range of REX-GECO1, which could elevate the field performance of red biosensors with broader applications for calcium-sensing into deep tissues and away from the bluer autofluorescence.

## 2. Materials and Methods

### 2.1. Protein Sample Preparation and Steady-State Electronic Spectroscopy

The detailed protein purification steps were reported in prior reports [18,23,27,31]. Following the protein extraction and purification by Ni-NTA affinity chromatography (MCLAB), the protein samples in a visually transparent buffer solution were used as is. To measure the steady-state electronic properties of REX-GECO1 at several pHs (3.5, 7, and 11), *in vitro* buffer solutions were prepared that contained 30 mM trisodium citrate, 30 mM borax, and 10 mM EGTA (for the Ca^2+^-free biosensor sample) or Ca^2+^-EGTA (for the Ca^2+^-bound biosensor sample). The concentrated REX-GECO1 proteins were then added and diluted into the various buffers to achieve µM concentrations under specified pH conditions.

The steady-state electronic absorption measurements were performed using Beckman DU-800 (Campbell Lab [18]) and Thermo Scientific Evolution 201 UV/Visible (Fang Lab [18,31]) spectrophotometers. The fluorescence data were taken using a Shimadzu RF-6000 spectrofluorophotometer. In particular, the fluorescence spectra were measured using a 3D-scan feature where the excitation and emission wavelengths were simultaneously tuned. The excitation wavelengths were scanned at 5.0 nm intervals from 360–650 nm and the emission wavelengths spanned from 370–800 nm at 1.0 nm intervals. The excitation and emission slices were taken from these scans and presented below and Figure S1 in the Supplementary Materials. The spectral scan speed was set at 2000 nm/min. For the fluorescence measurements, the protein sample concentration was diluted to ~10 µM to avoid self-reabsorption (inner filter effect [39]) when housed in a four-sided quartz cuvette with a 5 mm pathlength. The excitation and emission slit widths were both set to 3.0 nm for the Ca^2+^-free and -bound biosensor samples, while the corresponding detector sensitivity was set to high and low due to the rather weak and relatively strong emission intensities, respectively. All the spectroscopic experiments were conducted at ambient pressure (1 atm) and room temperature (~22 °C or 295 K).

### 2.2. Femtosecond Transient Absorption and Femtosecond Stimulated Raman Spectroscopy

A detailed description of our home-built optical setup has been reported previously [40,41,42,43]. Our optical setup begins with a Ti:sapphire regenerative amplifier (Legend Elite-USP-1K-HE, Coherent, Inc., Santa Clara, CA, USA) that generates a ~35 fs laser pulse centered at 800 nm with an operating average power of ~3.7 W at 1 kHz repetition rate. The 400 nm actinic pump pulse used as the excitation source for the fs-TA measurements was generated by focusing a portion of the laser fundamental output onto a beta-barium-borate (BBO) crystal via second harmonic generation (SHG). The temporal compression was achieved via a UV-grade fused silica ultrafast Brewster-angle dispersing prism pair (06SB10, Newport, Inc., Irvine, CA, USA) for a pulse duration below 100 fs, resulting in a cross-correlation time of ~140 fs for all the fs-TA and FSRS measurements [43]. The probe used for fs-TA measurements was supercontinuum white light generated by focusing a portion of the 800 nm fundamental pulse train onto a deionized-water-filled quartz cuvette with a pathlength of 2 mm. The transmitted probe was directed into an imaging spectrograph (IsoPlane SCT-320, Princeton Instruments, Inc., Trenton, NJ, USA) that disperses the probe onto a CCD array camera (PIXIS:100F, Princeton Instruments, Inc.) at the exit focal plane.

The ground-state FSRS measurements were performed with the Raman pump wavelengths of 650 and 800 nm. The conventional 800 nm Raman pump (R_pu_) with ~2 ps pulse duration was generated from the fs fundamental pulse centered at 800 nm, through a grating-slit-based spectral filter, which selects a portion of the 800 nm fs pulse and effectively produces a narrowband R_pu_ [44]. To generate the tunable R_pu_ centered at 650 nm, an fs noncollinear optical parametric amplifier (NOPA) was used to produce the fs seed pulse at the desired wavelength. Following a spectral filter that converts the fs seed into a ps seed pulse, a two-stage NOPA was used to amplify the ps Raman pump to an appreciable power necessary for the FSRS measurements [40]. To generate the tunable actinic pump for the excited state (ES)-FSRS measurements, a two-stage fs NOPA is required to amplify the actinic pump power at the desired wavelength (480 nm in this work) as the photoexcitation source. For the ground state (GS)-FSRS measurements, the actinic pump was blocked while the R_pu_ was chopped at half of the laser repetition rate (500 Hz) so one Raman spectrum can be obtained in 2 ms [44]. For ES-FSRS data collection, an electronic shutter was placed in the actinic pump beam path. The white-light probe for FSRS was generated in the same way as the fs-TA setup, and the cross-correlation time of the setup was ~140 fs. For both fs-TA and FSRS experiments, the incident laser pulses were overlapped spatially and temporally onto the 1-mm-thick cuvette housing the sample, prior to the transmitted probe being focused into the spectrograph. For the FSRS data collected on the Stokes and anti-Stokes sides [37,42], the Raman probe wavelengths are redder and bluer than the R_pu_ peak wavelength, respectively.

Moreover, for fs-TA and FSRS measurements, the reflective grating inside the spectrograph was set to 300 grooves/mm with a 300 nm blaze wavelength and 1200 grooves/mm with a 500 nm blaze wavelength, respectively. For the fs-TA experiments with a 400 nm actinic pump, the pump power was set to ~0.3 mW. For the GS-FSRS measurements with an 800 nm R_pu_, the R_pu_ power was set to ~2 mW [43]. For the GS/ES-FSRS with a 650 nm R_pu_ and a 480 nm actinic pump, the respective laser average powers were ~2 and 0.3 mW (GS-FSRS data were collected periodically throughout the ES-FSRS data collection by blocking the actinic pump) [43,45]. The protein biosensor sample’s optical densities (ODs) for the fs-TA and FSRS measurements were set at ~0.5 and 1/mm, respectively. Based on the protein biosensor’s peak ODs and pathlength of the cuvette, we can estimate the sample concentrations to be ~250 and 500 μM for the fs-TA and FSRS measurements, respectively. The sample solution was constantly stirred via a home-made magnetic stir bar to ensure a fresh protein solution sample was irradiated by the laser pulse train during the ultrafast spectroscopic measurements. The protein sample integrity was checked by taking the UV/Visible absorption spectra before and after each ultrafast spectroscopic measurement, commonly resulting in a spectral variation within 5% under the typical laser irradiation conditions used in our fs-TA and FSRS experiments [38,43].

### 2.3. Quantum Chemistry Calculations and Molecular Modeling

Ground state calculations of the Met223-Tyr224-Gly225 (MYG) chromophore within the REX-GECO1 biosensor were performed using the Gaussian 16 software [46]. The deprotonated chromophore structures and energies were specifically optimized using the density functional theory (DFT) method with the RB3LYP functional and 6-31G+(d,p) basis sets *in vacuo*. The same level of theory and basis sets were used in the vibrational normal mode frequency calculation to determine the ground-state Raman spectra. The chromophore was capped with methyl groups at the two ends (see Figure S6b,c in the Supplementary Materials) to mimic the chromophore embedded in a protein pocket [43,47,48,49,50].

The PyMOL software [51] was used to model the chromophore pocket of REX-GECO1 based on the crystal structure of R-GECO1 (PDB: 4I2Y) (20) that was the progenitor for the successfully engineered REX-GECO1 biosensor to undergo the Ca^2+^-dependent ESPT reaction [27]. The Lys80Glu mutation was then performed based on the R-GECO1 crystal structure using the mutagenesis wizard within PyMOL software to model and visualize key structural parameters involving the RFP chromophore. No energy minimization (e.g., by molecular dynamics simulations) was performed after the *in silico* mutation, which can inspire future investigations of pertinent structural variations due to all 14 mutations in REX-GECO1 relative to R-GECO1 [27] with advanced computational methods.

## 3. Results and Discussion

### 3.1. Steady-State Electronic Spectroscopy of REX-GECO1 Reveals Influence of a Nearby Amino Acid on the Chromophore’s Electronic Properties

**Figure 1:**
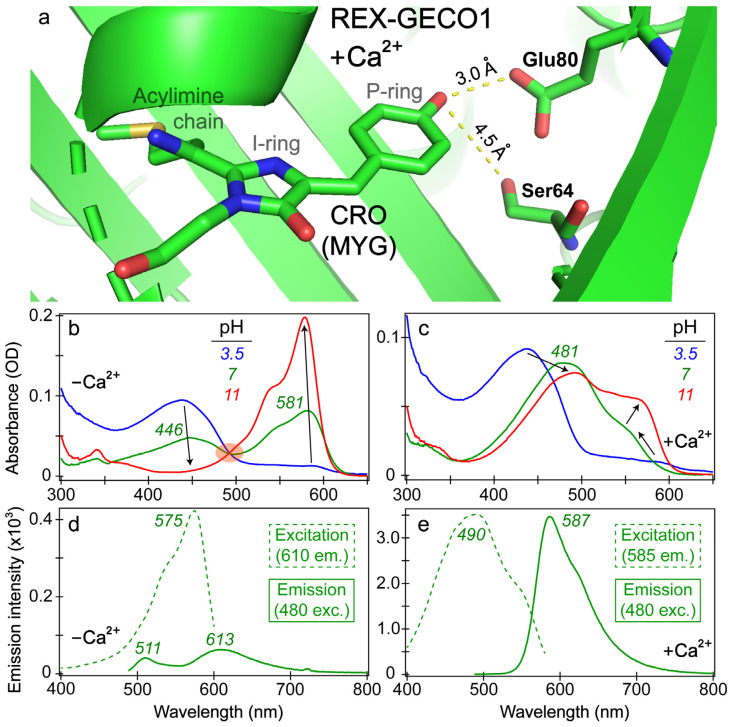
Visualization of the REX-GECO1 chromophore pocket and steady-state electronic spectra. (**a**) Chromophore (CRO) and key residues inside the Ca^2+^-bound REX-GECO1 biosensor, modeled using the progenitor R-GECO1 crystal structure in the Ca^2+^-bound state (PDB ID: 4I2Y) [20]. Interactions between the chromophore phenolic end and key surrounding residues are highlighted by dashed yellow lines, with the distances labeled in angstrom units. Distinct chromophore P- and I-ring moieties and the acylimine chain at the I-ring end are labeled. Steady-state absorption spectra of the (**b**) Ca^2+^-free and (**c**) Ca^2+^-bound REX-GECO1 biosensor are shown at pH values of 3.5 (blue), 7 (green), and 11 (red). Black arrows denote major peak changes with the pH increase. The shaded orange ellipse in panel (**b**) highlights the isosbestic point as the pH varies. The excitation (dashed) and emission (solid) spectra of the (**d**) Ca^2+^-free and (**e**) Ca^2+^-bound biosensors are shown in pH 7 buffer with the excitation profiles recorded at the emission wavelengths of 610 and 585 nm, respectively. The emission spectra were both collected upon 480 nm excitation of the biosensor.

To better understand the effects of calcium binding on the photophysical properties of REX-GECO1, we inspected the chromophore pocket of the Ca^2+^-bound biosensor (Figure 1a). Without a currently available REX-GECO1 crystal structure, we modeled the local environment using the crystal structure of the parent biosensor, R-GECO1 in the Ca^2+^-bound state [20,27]. During the prior development of an excitation-ratiometric biosensor from R-GECO1, three residues (Ser64, Lys80, and Ile116 using the GCaMP numbering in PDB ID 3EVR [52] protein) in proximity to the chromophore’s phenolic or phenolate ring (depending on the protonation state) were identified as key mutation sites to improve the protein biosensor performance [27]. After a series of screenings and modifications, the best-performing REX-GECO1 biosensor has 14 mutations relative to R-GECO1, including the crucial Lys80Glu mutation. The spatially close glutamic acid not only stabilizes the protonated chromophore via direct electrostatic interaction but also acts as a proton acceptor upon light activation of the protonated chromophore, thus leading to a large Stokes shift of the biosensor fluorescence. Upon Ca^2+^ binding, the protein biosensor’s CaM subunit undergoes conformational changes to wrap around the M13 peptide [27,29]. This conformational rearrangement compresses the cavity volume of the linked RFP subunit, reducing space between the three-residue (MYG) chromophore and an adjacent Glu80 residue inside the biosensor. In contrast, without calcium ions, the RFP cavity size is larger while Glu80 is directed away from the photosensory active site (hence hindering ESPT from the chromophore on ultrafast timescales and reducing red emission of the biosensor, see below) as summarized in prior studies [18,27].

The increased/decreased interactions between the RFP chromophore and Glu80 in the Ca^2+^-bound/free biosensors exert marked effects on steady-state spectroscopic profiles (see Figure 1b–e). The absorption profile of the Ca^2+^-free biosensor (Figure 1b) displays the prototypical behavior of a two-state model as the aqueous buffer pH increases from low (pH 3) to high (pH 11) values, evidenced by a clear isosbestic point. At a neutral buffer (pH 7), both the protonated and deprotonated chromophore populations are appreciably populated with peak absorbance wavelengths of ~446 and 581 nm, respectively. The Ca^2+^-bound biosensor displays a more nuanced change of the absorption profile as the pH increases with no clear isosbestic point (Figure 1c). At low pH, the absorption profiles of REX-GECO1 are similar with and without calcium ions; however, at neutral pH, the Ca^2+^-bound biosensor red-shifts its absorption peak from ~437 to 481 nm. This newly formed state with a notably red-shifted absorption peak was assigned to a distinct orientation/configuration of the protonated chromophore relative to the adjacent Glu80 residue, which has a typical p*K*_a_ value of ~4.25 [18]. A systematic pH-dependent evaluation of the REX-GECO1 biosensor revealed that the redshift of the protonated Ca^2+^-bound peak absorption occurs around buffer pH 5, suggesting that the deprotonation of the nearby Glu80 can effectively lead to a redshift of the protonated MYG chromophore’s absorption peak wavelength, in accord with stabilization of the neutral phenol with a higher p*K*_a_ than 4.25 [27]. For corroboration, quantum calculations show that the protonated chromophore’s S_0_→S_1_ energy gap (e.g., vertical excitation) significantly red-shifts with a nearby deprotonated Glu80 residue (with a carboxylate), in comparison to that with a nearby protonated Glu80 residue (with a carboxylic acid) [18]. These results provide strong evidence that the dominant absorption peak redshift of the Ca^2+^-bound biosensor in buffer pH 3.5 to 7 solutions reflects the protonated chromophore (A form) population, which displays an altered p*K*_a_ and energy gap due to a key proximal charged residue. As the pH continues to increase from 7 to 11, the Ca^2+^-bound biosensor’s spectral profile continues to change with a prominent deprotonated chromophore (B form) absorption peak growing in at ~560 nm and shifting to 566 nm (Figure 1c).

Importantly, the Glu80 residue affects the fluorescence properties of the Ca^2+^-bound biosensor as well, manifested by the comparison between the Ca^2+^-free versus -bound emission peaks (see Figure 1d,e). In the latter, a dominant emission peak assigned to the deprotonated chromophore can be observed at 587 nm upon 480 nm excitation of the protonated chromophore, resulting in a Stokes shift over 100 nm (~0.47 eV). When monitoring the emission at 585 nm, the excitation peak maximum is 490 nm, suggesting that the red emission from the deprotonated chromophore is primarily due to ESPT that originates from the protonated chromophore (A form). This result is consistent with the general mechanism of a large Stokes shift RFP [53]. Notably, a small shoulder at the red edge of the excitation peak maximum can be observed near 550 nm (see Figure 1e), corresponding to the electronic ground state (S_0_) of the deprotonated chromophore (B form) with a greatly reduced contribution to the overall red fluorescence of the biosensor.

In contrast, the Ca^2+^-free biosensor’s emission spectrum features an interesting dual-emission spectral profile after 480 nm excitation with the peak maxima at 511 and 613 nm, associated with the protonated and deprotonated chromophores, respectively [18]. The Stokes shift is smaller (0.35 eV) for the protonated form and larger (0.76 eV) for the deprotonated form (both with respect to the A form absorption peak at 446 nm, Figure 1b) when compared to the sole emissive state of the Ca^2+^-bound biosensor (0.47 eV, see above). Such a large Stokes shift is relatively uncommon (with one comparable RFP called mKeima [53,54,55]), even for ESPT-capable chromophores, suggesting that the apparent weak fluorescence at 613 nm may result from direct excitation of the deprotonated chromophore (B form with an absorption peak at 581 nm), which has a substantial population at pH 7 (green trace in Figure 1b). In addition, when setting the emission detector at 610 nm, the excitation spectrum of the Ca^2+^-free biosensor primarily resembles the deprotonated chromophore absorption spectrum (λ_max_ = 581 nm) with an excitation peak maximum at 575 nm, along with little contribution from the protonated chromophore that absorbs below 500 nm (green dashed line in Figure 1d, see below for more discussions). The steady-state spectral analysis indicates that the compressed RFP cavity size inside the Ca^2+^-bound biosensor reduces the distance between the chromophore P-ring and nearby Glu80 versus the more spacious chromophore pocket in the Ca^2+^-free REX-GECO1. The strengthened chromophore-Glu80 polar interaction (electrostatic in nature) leads to a much more prominent ESPT for the Ca^2+^-bound biosensor with the negatively-charged Glu80 as the proton acceptor, evidenced by the large Stokes shift of the emission spectrum upon 480 nm excitation (Figure 1e) and the prominent contribution from the protonated chromophore (A form) leading to 587 nm emission from the deprotonated chromophore (i.e., I* form that represents the light-induced ESPT product, highlighting its unrelaxed protein environment in the excited state versus B form in a relaxed protein environment in the ground state) [56,57,58]. In particular, the interatomic distance (Figure 1a) supports the H-bonding interaction between the phenolic hydroxy group of the MYG chromophore and the deprotonated form of Glu80 at neutral pH, constituting an essential part of the ESPT pathway.

While the contribution from the protonated chromophore (A form) at the deprotonated form emission wavelength (613 nm) is greatly reduced for the Ca^2+^-free biosensor (Figure 1d), there is still an appreciable emission intensity at wavelengths above 600 nm upon 480 nm excitation. We reckon there are three possible explanations for this red fluorescence peak: (1) direct excitation of the deprotonated chromophore (B form) so B* emits at 613 nm, (2) FRET from the A* form emission (donor, the asterisk denoting the excited state) to the B form absorption (acceptor), and (3) ESPT of the A* form to generate I* form (see above). The direct excitation model holds some merit by observing the emission spectra with bluer excitations than 480 nm so a broader spectral coverage provides more insights [38,43,59,60]. In particular, 480 nm light can excite the red shoulder of the A form absorption peak while achieving some direct excitation of the blue edge of the B form absorption band (Figure 1b). Interestingly, the emission intensity from A* is significantly less intense at this excitation wavelength than B* emission, as shown by the green solid trace in Figure 1d and the blue solid trace in Figure S1b. For comparison, the Ca^2+^-free biosensor’s emission spectra after 400 and 440 nm excitation (Figure S1) show that the bluest excitation (400 nm in this work) enables the A* emission peak (~511 nm) to be more intense than the B* emission peak (~613 nm, assuming direct excitation of B form is still feasible, see below). With the “intermediate” 440 nm excitation, the two emission peaks are essentially of equal intensity. This observed trend, where the deprotonated form emission peak becomes relatively more intense as the excitation wavelength increases (400 → 440 → 480 nm), supports the direct excitation model.

On the other hand, the observed trend could be further explained and/or contributed by structural inhomogeneity. For example, if ESPT from the protonated chromophore was the reason for the deprotonated form emission peak, then bluer excitations (400 and 440 nm) could excite a subpopulation that is less prone to undergoing ESPT (e.g., a stronger phenolic –OH bond or a local environment with no prominent proton acceptors [38,61,62]), whereas the redder excitation (480 nm) may excite a subpopulation that is more prone to undergoing ESPT. Such a specific explanation involves many assumptions, which require further experimental support, motivating future studies on the detailed multidimensional excited-state potential energy landscape of protein chromophores driven by light [38,59,63,64]. With regards to the FRET model, a similar inhomogeneity argument could be made where 400 nm light excites a protonated subpopulation with a reduced FQY or a protonated subpopulation that emits at bluer wavelengths (thus with less spectral overlap between the donor emission and acceptor absorption bands).

Since the peak wavelengths and spectral profiles of the A* emissive state remain identical regardless of the excitation wavelength (see Figure S1b), we can then essentially eliminate these FRET-based interpretations. Moreover, while the A* emission peak is less intense upon 400 or 440 nm excitation compared to 480 nm excitation, the deprotonated form emission peak (from B* and/or I*) becomes much weaker with bluer excitations. In other words, if FRET constitutes the main reason for the apparent B* emission, the A* and B* emission intensities should display a correlated change, which does not match the observed larger increase of B* emission peak than that of A* when going from 440 to 480 nm excitation (Figure S1b). One caveat is that the excitation-dependent chromophore inhomogeneity could be masked by FRET. For example, 480 nm light may excite a subpopulation that emits at redder wavelengths and undergo FRET to a larger extent, leading to a more intense 613 nm peak. In contrast, 400 or 440 nm light does not excite this subpopulation to a similar degree, hence showing a reduced emission peak at 613 nm. Nevertheless, such postulates speak to the complexity of an RFP-based chimeric protein biosensor, while we prefer to focus on the major fluorescence properties with simpler yet sufficient models. Our time-resolved electronic spectroscopic results can shed some important light based on the ultrafast dynamics of the relevant B*/I* stimulated emission features (see Section 3.2 below).

Another relevant explanation for the direct excitation of B form leading to the B* emission peak (i.e., an “add-on” to the primary interpretation (1) as stated above) is that 400–480 nm excitation could excite the ground-state B population to higher-lying excited states (e.g., S_2_, S_3_) instead of just S_1_. Upon inspection of the Ca^2+^-free biosensor’s absorption spectral profile at pH 11, there remains some absorption component at wavelengths below 480 nm (Figure 1b). However, according to this interpretation, 480 nm excitation should lead to the most intense deprotonated form emission (OD = 0.018 at 480 nm), while 400 nm excitation should lead to the second most intense deprotonated form emission (OD = 0.007 at 400 nm) with 440 nm excitation having the least intense deprotonated form emission (OD = 0.005 at 440 nm). Instead, we observed that the deprotonated form emission intensity pattern follows the trend with excitation wavelengths: 480 nm > 440 nm > 400 nm in pH 7 buffer (i.e., the 440 and 400 nm cases are reversed, see Figure S1). Considering all these robust observations by steady-state electronic spectroscopy, it seems that the FRET-based interpretation is the least likely, while the direct excitation (from B form in S_0_ to B* form in S_1_) and ESPT (from A* to I* forms in S_1_) models hold strong merit without invoking excessive assumptions about the A and B forms of the protein chromophore at neutral pH conditions.

### 3.2. Femtosecond Transient Absorption Exposes the Dark-State Formation and Excited-State Proton Transfer in the Ca^2+^-Free and -Bound Biosensors

**Figure 2:**
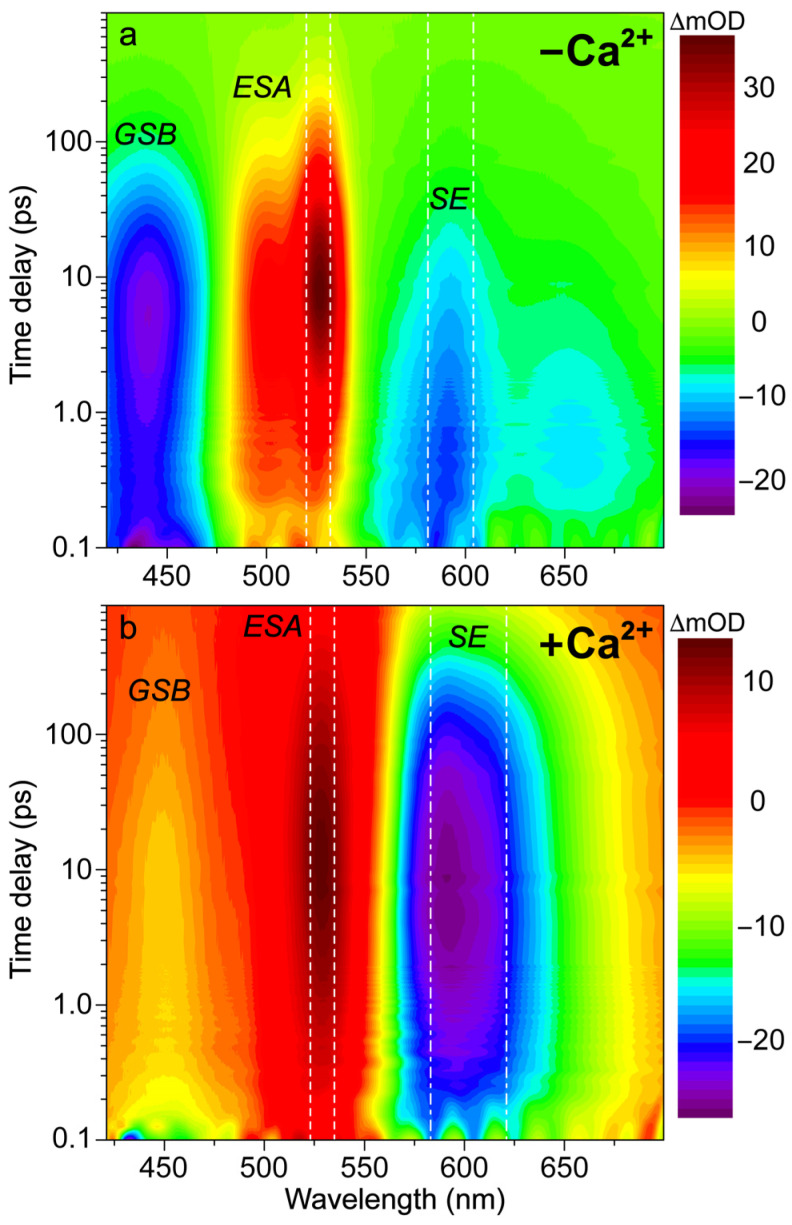
Contour plots of fs-TA spectra of REX-GECO1 biosensor in pH 7 buffer (**a**) without and (**b**) with calcium ions. Upon 400 nm excitation, the Ca^2+^-free (−Ca^2+^) and -bound (+Ca^2+^) biosensors display transient electronic features including ground-state bleaching (GSB), excited-state absorption (ESA), and stimulated emission (SE) bands over the experimental time window spanning 900 ps. The signal intensity is shown by the color bar on the right side of each panel in the unit of milli-optical density (mOD). The vertical white dashed or dash-dotted lines depict the spectral integration region for the TA probe-dependent intensity dynamics presented below.

In order to better explain the appearance of the deprotonated emission peak under near-UV to visible light, we performed fs-TA experiments on the Ca^2+^-free and -bound REX-GECO1 biosensors with 400 nm excitation to compare to the previously reported 480 nm excitation results [18]. In addition to achieving a more exclusive excitation of the A form with 400 versus 480 nm light (i.e., on the blue edge versus red edge of the A form absorption band, see Figure 1b), the bluer excitation with a wider spectral window allows for analysis of the photoexcited protonated form’s ground-state bleaching (GSB) for both the Ca^2+^-free and -bound biosensors as well as a potential protonated (A*) form’s stimulated emission (SE) band. In Figure 2, the fs-TA spectral contour plots of the Ca^2+^-free and -bound REX-GECO1 exhibit primarily three features following 400 nm excitation: GSB from ~420–475 nm, excited-state absorption (ESA) from ~475–550 nm, and SE from ~550–700 nm. For the Ca^2+^-free (Figure 2a) and Ca^2+^-bound (Figure 2b) cases, it is readily apparent that the deprotonated form’s SE band is much stronger when the biosensor is bound to calcium ions. This observation alone indicates that the Ca^2+^-bound biosensor undergoes ESPT to a much larger degree than the Ca^2+^-free counterpart even when considering the possibility of direct excitation of B form because there is a greatly reduced deprotonated chromophore (B) population at neutral pH for the Ca^2+^-bound biosensor (Figure 1c) than the Ca^2+^-free biosensor (Figure 1b).

With regards to the potential for direct excitation explaining the prompt appearance of the deprotonated SE for the Ca^2+^-free biosensor, a comparative analysis of the fs-TA spectra upon 400 versus 480 nm excitation can provide several illuminating insights. With 480 nm excitation [18], the absolute SE peak magnitude reaches a maximum intensity of ~18 mOD, while the corresponding intensity upon 400 nm excitation is ~14 mOD. The smaller magnitude of the deprotonated SE band (PB*) after 400 nm excitation is similar to the trend observed with the excitation-dependent steady-state fluorescence spectra (see Figure S1), in accord with the aforementioned direct excitation model. Moreover, the adjacent ESA band to the blue side (470–550 nm) reaches a maximum intensity of ~28 mOD upon 480 nm excitation compared to ~36 mOD with 400 nm excitation (Figure 2a). Upon comparison of the maximum intensities of the SE to the ESA band, the relative intensity ratio upon 400 nm excitation is ~39% ($\frac{SE\;peak\;magnitude\;at\;300\;fs}{ESA\;peak\;intensity\;at\;10\;ps}=\frac{14\;mOD}{36\;mOD}$) versus ~64% ($\frac{SE\;peak\;magnitude\;at\;300\;fs}{ESA\;peak\;intensity\;at\;10\;ps}=\frac{18\;mOD}{28\;mOD}$) following 480 nm excitation. Interestingly, by comparing the SE peak intensity magnitude upon 400 versus 480 nm excitation, the relative intensity ratio is ~78% ($\frac{SE\;peak\;magnitude\;upon\;400\;nm\;exc.}{SE\;peak\;magnitude\;upon\;480\;nm\;exc.}=\frac{14\;mOD}{18\;mOD}$) while the ESA relative intensity ratio is the opposite ($\frac{ESA\;peak\;intensity\;upon\;480\;nm\;exc.}{ESA\;peak\;intensity\;upon\;400\;nm\;exc.}=\frac{28\;mOD}{36\;mOD}\approx 78\%$). Such opposite intensity ratios suggest that spectral overlap affects the apparent TA features of the Ca^2+^-free REX-GECO1 (i.e., a larger SE upon 480 nm excitation leads to a smaller ESA and vice-versa); however, this correlation also indicates that the ESA originates primarily from a different electronic excited state than the SE. If this ESA were to arise from the deprotonated chromophore only, one would expect the ESA to be larger upon 480 nm excitation because the B* SE is larger, representing an increased chromophore population reservoir to funnel to the proposed dark state [18]. Hence, we deduce that A* significantly contributes to the ESA band while the B* SE band promptly rises due to the direct excitation of B in the Ca^2+^-free REX-GECO1 biosensor (Figure 2a).

However, the possibility for an ultrafast ESPT reaction from the protonated chromophore in the Ca^2+^-free biosensor within the cross-correlation time of our laser optical setup [65] cannot be excluded and may play a minor role. Similar to the discussion revolving around the excitation-dependent steady-state fluorescence profiles (Figure 1d and Figure S1), the chromophore inhomogeneity could explain the apparent mismatch between the relative SE and ESA intensities, which slightly disrupts the interpretation that the Ca^2+^-free biosensor’s dark-state formation primarily originates from the A* population. We note that the term “dark state” generally refers to an electronic excited state (S_1_) with an extremely low oscillator strength for the S_1_→S_0_ radiative transition in these FPs. For example, one could argue that both 400 and 480 nm excitations lead to ESPT to generate I*, but 480 nm excitation forms a more planar I* chromophore (hence an enhanced I* SE and reduced dark-state ESA), whereas the excess energy with 400 nm excitation leads to a more twisted I* state (hence a smaller deprotonated form SE and larger dark-state ESA). Nevertheless, FRET remains a highly unlikely interpretation for the prompt rise of the deprotonated SE because FRET typically occurs on the ps-to-ns timescales for most FPs [21,45,66,67,68], which is significantly longer than the sub-140 fs timescale necessary to rationalize the prompt SE appearance at the photoexcitation time zero.

This seemingly straightforward discussion is relevant for improving the dynamic range of the REX-GECO1 biosensor since it is imperative to understand the origin of the observed deprotonated form emission after 400 nm excitation of the Ca^2+^-free biosensor. In particular, if ESPT is the dominant contributor, rational design strategies need to be invoked to reduce the ESPT capability because red emission is detrimental to ratiometric enhancement in the absence of calcium ions. On the other hand, if the deprotonated form SE is due to direct excitation of B, rational design strategies need to be devised to enlarge the spectral separation between the A and B absorption peaks at neutral pH, especially with 480 nm excitation where the ratiometric measurements are most relevant at physiological pH [18,23,26]. A relatively simple experiment may address this debacle: performing the fs-TA experiment with 400/480 nm excitation on the Ca^2+^-free REX-GECO1 at low pH (e.g., pH 3.5). At such a low pH, there is essentially no B form population (Figure 1b). Therefore, if the deprotonated form SE band was still observed at an appreciable intensity, the only rational explanation would be ESPT (i.e., A*→I*). In contrast, if the deprotonated form SE was not observed, then direct excitation of the B form at neutral pH could be a notable contributor. While this hypothetical experiment is sound in theory, it was not practical experimentally due to the instability of the protein biosensor at such a low pH over the ~2 h time window required for conducting the complete fs-TA measurements. For potential future investigations, possible mitigation strategies could involve the set-by-set collection and analysis of fs-TA data since, for each set scanning, the experimental time window of a freshly prepared protein sample takes ~20 min (although it would require much more proteins in total), as well as the search for more suitable buffer solutions to stabilize the protein biosensor at this acidic pH.

**Figure 3:**
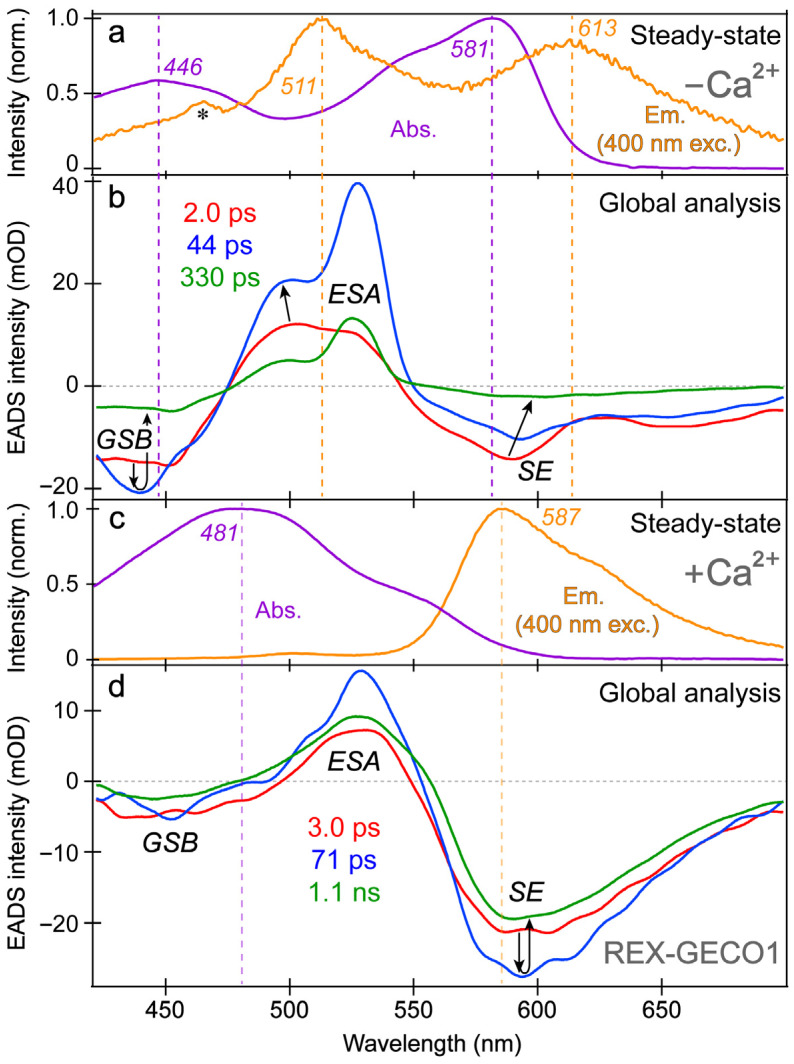
Steady-state electronic spectroscopy and global analysis of the fs-TA spectra of REX-GECO1 in pH 7 aqueous buffer. The steady-state absorption (purple) and emission (orange, with 400 nm excitation) spectra are displayed for the (**a**) Ca^2+^-free and (**c**) Ca^2+^-bound REX-GECO1, respectively. Major peaks are labeled. The asterisk in (**a**) marks a small water scattering peak on top of the weak fluorescence profile. The sequential evolution-associated difference spectra (EADS, red→blue→green) of the (**b**) Ca^2+^-free and (**d**) Ca^2+^-bound REX-GECO1 are shown with the retrieved excited state lifetimes (color-coded) after 400 nm excitation. The vertical dashed lines highlight the steady-state peak wavelengths with respect to transient spectral features, while the black arrows in (**b**,**d**) highlight the spectral evolution in GSB, ESA, and SE regions.

To provide further insights into the SE band origin of the Ca^2+^-free biosensor in Figure 2a, several additional experimental observations can be made. The SE-to-ESA intensity ratio at 300 fs time delay is much lower following 400 nm excitation ($\frac{SE\;peak\;magnitude\;at\;300\;fs}{ESA\;peak\;intensity\;at\;300\;fs}=\frac{14\;mOD}{14.5\;mOD}\approx 96\%$) than that after 480 nm excitation ($\frac{SE\;peak\;magnitude\;at\;300\;fs}{ESA\;peak\;intensity\;at\;300\;fs}=\frac{18\;mOD}{13\;mOD}\approx 138\%$) [18], which again supports the direct excitation model as the primary cause of the prompt appearance of the deprotonated form SE band because 400 nm light is much less efficient at exciting the B form than 480 nm light. However, this observation could still be explained by the A form population inhomogeneity. The final verdict regarding these dueling interpretations is that the rise of the ESA peak is less significant upon 480 nm excitation ($\frac{ESA\;peak\;intensity\;at\;10\;ps}{ESA\;peak\;intensity\;at\;300\;fs}=\frac{28\;mOD}{13\;mOD}\approx 215\%$) as reported [18] than that upon 400 nm excitation ($\frac{ESA\;peak\;intensity\;at\;10\;ps}{ESA\;peak\;intensity\;at\;300\;fs}=\frac{36\;mOD}{14.5\;mOD}\approx 248\%$) in this work (see Figure 2a). This finding indicates that the ESA rise is not directly related to the dark-state formation originating from the deprotonated chromophore because there is more B* immediately following 480 nm excitation (a larger SE band after time zero), yet the rise magnitude is clearly reduced. In other words, with a larger B* reservoir to access by 480 nm excitation, the dark-state ESA band rise is diminished, inferring that the dark state mainly originates from the A* form (protonated chromophore). For comparison, using 400 nm light to primarily excite the A form, a larger rise of the prominent ESA band is observed due to the more targeted excitation of the protonated chromophore (using the blue-edge excitation at 400 nm versus the red-edge excitation at 480 nm, see Figure 1b for the absorption spectrum of the Ca^2+^-free biosensor in pH 7 aqueous buffer). The larger rise is also likely affected by the more prominent decay of the protonated GSB upon 400 nm excitation (benefited from a probe window that extends as blue as ~420 nm in this work, see Figure 2 and Figure 3), evidenced by the much greater rise of the blue ESA component (see Figure 3b and Figure S2, the ESA band’s shoulder that is centered around 500 nm) versus the analogous rise upon 480 nm excitation [18]. Furthermore, the intrinsic anharmonicity of potential energy surfaces of the protein chromophore suggests that the ESA band should originate from A* (i.e., the ESA peak should be redder than the ground-state absorption or GSA peak that is ~446 nm for A form at pH 7) and not from B* (i.e., the ESA peak should not be bluer than the GSA peak that is ~581 nm for B form at pH 7).

The spectral overlap of the fs-TA transient features, especially for the Ca^2+^-free biosensor chromophore at neutral pH (Figure 3a,b), requires global analysis [69,70,71] to dissect the energy dissipation pathways following 400 nm excitation, supporting the aforementioned discussions of distinct electronic features (mainly the ESA and SE) and their dynamics. In contrast, for the Ca^2+^-bound biosensor (Figure 3c,d), a sequential model in global analysis yields the evolution-associated difference spectra (EADS), which display both ESA and SE features with visually similar dynamics, including a ~3 ps time constant (red→blue trace in Figure 3d). This process is assigned to ESPT upon excitation of the protonated chromophore (A form) due to the similar position and lineshape of the SE compared to the spontaneous fluorescence (Figure 3c). As shown in global analysis (Figure 3d) and 2D-contour plot (Figure 2b), the deprotonated form SE feature promptly rises due to an ultrafast ESPT within the cross-correlation time of the instrument because there is minimal deprotonated population at pH 7 (Figure 1c), although direct excitation of the deprotonated chromophore may still occur to a minor degree in this biosensor state. Following the concomitant rise of the ESA/SE bands, both transient spectral features decay with an ~71 ps time constant (blue→green trace in Figure 3d) assigned to nonradiative relaxation, likely including some conformational motions of the chromophore, which are coupled to the local environment rearrangement [38,72]. The final time constant retrieved from global analysis of the Ca^2+^-bound chromophore is 1.1 ns, portraying the apparent fluorescence lifetime of the chromophore and is typical for moderately fluorescent FPs [38,59,73].

Though we have extensively used the fs-TA technique in conjunction with global analysis to reveal transient electronic dynamics of various FPs and FP-based biosensors [38,43,45], a few details about the strategies and practices in this work can be provided. Three components were deemed sufficient by comparing the experimental data points to the exponential fits with two-, three-, four-, and five-component models. For the Ca^2+^-bound biosensor’s fs-TA dynamics (Figure 2b), a two-component model did not converge, suggesting its inadequacy to fit the entire spectral window with only two components. On the other hand, a four- and five-component model did not add any physically meaningful spectral features. For the four-component model, the lifetime of the first state was <60 fs in an attempt to fit the coherent artifact. For the five-component model, the lifetimes of the first two states were ~10 and 30 fs, again attempting to overfit the coherent artifact. While the four/five-component models did reduce the root-mean-square error (RMSE) of the fit (0.00111/0.00099) versus the three-component model (0.00127), we consider such a marginal reduction to be insignificant. A similar systematic evaluation of the Ca^2+^-free biosensor’s fs-TA dynamics (Figure 2a) was performed with a similar conclusion, and a similar process was followed for the ES-FSRS data (see Section 3.3 below). In each case, the sequential EADS model was favored over the parallel decay-associated difference spectra (DADS) model given the *a priori* knowledge of REX-GECO1 biosensor in addition to previous experiments and publications [18,27], and current spectral data. The output of such systematic analysis includes characteristic spectral profiles of the electronic features from both EADS (Figure 3b,d) and DADS with associated lifetimes. Furthermore, additional outputs include the RMSE comparing the fit to the experimental data, the full-width-at-half-maximum (FWHM) and time zero of the instrument response function, and other parameters used to correct for any spectral dispersion.

The SE and ESA dynamics of the Ca^2+^-free biosensor differ in that the sequential EADS reveal the SE decays while the ESA rises with a 2 ps time constant (Figure 3b, red→blue trace). In line with the previous reports on REX-GECO1 [18,27], the flexible and spacious chromophore pocket within the Ca^2+^-free biosensor allows the chromophore to twist rather freely upon photoexcitation, which is reflected by the 2 ps decay of the SE as some excited-state population nonradiatively returns to the ground state while the SE transition oscillator strength notably diminishes upon twisting. Moreover, the matching yet opposite decay/rise dynamics of the SE/ESA bands indicate that twisting of the deprotonated chromophore may contribute to the apparent rise of the ESA, a spectral signature of the dark state, yet its main contributor is the twisting of the protonated chromophore (see above).

At neutral pH, spectral overlap between the SE and ESA features inevitably complicates the spectral analysis (e.g., the SE decay can lead to the rise of an overlapped ESA if they originate from different states with different dynamics) [69]. Therefore, we assign the Ca^2+^-free biosensor’s ESA band to arise from an inhomogeneous mixture of the protonated and deprotonated chromophores that can twist on ultrafast timescales to reach a dark state. To provide additional evidence for direct excitation of the Ca^2+^-free biosensor’s deprotonated chromophore by 400 nm light, we observed pure decay dynamics at ~580 nm probe wavelength where the deprotonated chromophore (B form) mainly absorbs. If only ESPT occurred with no direct excitation of B form, we should not observe pure decay dynamics at 580 nm from time zero because no B form’s GSB band should be observed herein without B form excitation. Nevertheless, spectral overlap between the B form GSB band and B* form SE band may complicate this simplified interpretation, and there could be an ultrafast ESPT pathway that results in the observed broad SE band from a promptly emerged I* state, which then decays due to various energy relaxation pathways [37,38].

In addition, the negative feature to the blue side (Figure 3b, ~425–475 nm) could be assigned to originate from two states: SE from A* or GSB of A. We primarily assign this feature to the GSB of the A form because the emission intensity of the Ca^2+^-free biosensor is inherently weak, implying that the SE cannot contribute to the TA spectra as significantly when compared to absorption from the ground state with an appreciable oscillator strength. Furthermore, the peak wavelength of this transient negative feature is below 450 nm (Figure 3b), bluer than the spontaneous emission peak (Figure 3a). While bluer SE features have been observed in fs-TA spectra relative to the spontaneous fluorescence in FPs [38,43], such a blue-shifted SE feature with essentially no Stokes shift is unlikely. In fact, the peak of the feature (Figure 3b, blue trace) is even bluer than the ground state absorption, which confirms that the feature primarily originates from GSB with minor contributions from the A* SE at redder wavelengths (~450–500 nm that encompasses the blue side of the A* fluorescence peak at ~510 nm).

With this GSB assignment of the A form, a new question arises: why does this GSB band rise on the ~2 ps timescale (Figure 3b, red→blue traces)? A delayed GSB rise pattern after time zero is physically unreasonable unless the GSB is overlapped with a positive feature [74]. Without direct evidence, we speculate that this overlapping positive feature originates from the A* form S_1_→S_2_ or S_3_ transitions due to the proximity to the A form ground-state absorption wavelengths while considering an anharmonic potential energy surface [72,75]. This observation again indicates that the Ca^2+^-free biosensor does not undergo ESPT to a large degree. First, if the inferred decay of the positive ESA from A* were attributed to the A*→I* transition, then the I* SE should show a matching rise with an ~2 ps time constant instead of an initial decay (Figure 3b). Second, for a typical ESPT process, the A form’s GSB band should decay as the proton is transferred due to the nature of the GSB signal (i.e., a smaller ground-state population with the actinic pump on (85–90%) versus off (~100%) [57,76]). Notably, the Ca^2+^-bound biosensor’s GSB primarily shows a decay starting from time zero, which is consistent with a prominent A*→I* ESPT reaction (Figure 3d). Therefore, we assign the rise of the Ca^2+^-free biosensor’s protonated chromophore (A form) GSB band to the “overlapping” decay of the A* ESA band upon a characteristic P-ring twist with an ~2 ps time constant (Figure 3b), forming a dark state, which is apparent from the prominent rise of an adjacent ESA band on the red side (~475–550 nm). In short, the Ca^2+^-free biosensor’s ESA band primarily originates from a twisted, dark state of the A* form with some contributions from a similar dark state of the deprotonated chromophore (B* form).

Further evidence for the different origins of the ~2 ps decay (Figure 3b) and 3 ps rise (Figure 3d) of the deprotonated form SE band in the Ca^2+^-free and -bound REX-GECO1, respectively, can be gleaned from the observed ~2 ps redshift of the B* SE peak in the Ca^2+^-free biosensor (Figure 3b, red→blue trace). This redshift is not observed for the Ca^2+^-bound biosensor’s I* SE band (Figure 3d) and is thus assigned to the relaxation of the Ca^2+^-free biosensor’s excited state upon the chromophore twist, leading to a reduced energy gap between the singlet excited and ground states (S_1_→S_0_). The subsequent 44 ps lifetime in the Ca^2+^-free case tracks a pronounced decay of all the TA bands (Figure 3b, blue→green trace), likely corresponding to the ~71 ps decay component in the Ca^2+^-bound biosensor (Figure 3d, blue→green trace). The small-scale conformational motions and local environment rearrangement on this timescale occur to a much larger extent for the Ca^2+^-free biosensor, evidenced by the faster and much larger decay (Figure 3b) than the Ca^2+^-bound counterpart (Figure 3d). The initial redshift of the Ca^2+^-free biosensor’s SE peak is supplemented by a slight blueshift of the ESA peak (Figure 3b), indicative of additional relaxation of the S_1_ state on the intermediate (44 ps) timescale that is not observed within the more rigid Ca^2+^-bound biosensor, where no such shift is observed (Figure 3d). This comparison supports our previous findings that the Ca^2+^-free biosensor has a more flexible local environment that allows for faster and more prominent twisting of the chromophore than the compressed and more rigid local environment in the Ca^2+^-bound biosensor [18]. Furthermore, the red-shifted shoulder of the Ca^2+^-free SE feature (Figure 3b, ~630–700 nm as an electronic signature [72,77]) is more prominent than the Ca^2+^-bound counterpart, suggesting a twisted intramolecular charge transfer (TICT) state that may become especially prominent without calcium binding [77,78]. The final time constant (330 ps) of the Ca^2+^-free biosensor also supports this interpretation because the apparent fluorescence lifetime is much shorter than the Ca^2+^-bound counterpart (1.1 ns) [38,53]. The apparent fluorescence lifetime is an average of nonradiative and radiative time constants [77,79], the significantly shorter lifetime of the Ca^2+^-free biosensor is due to more prominent nonradiative transitions that are consistent with the more spacious local environment and reduced FQY (Figure 1d,e). This reduced FQY is directly related to the prominent dark-state formation in the Ca^2+^-free biosensor upon light irradiation.

**Figure 4:**
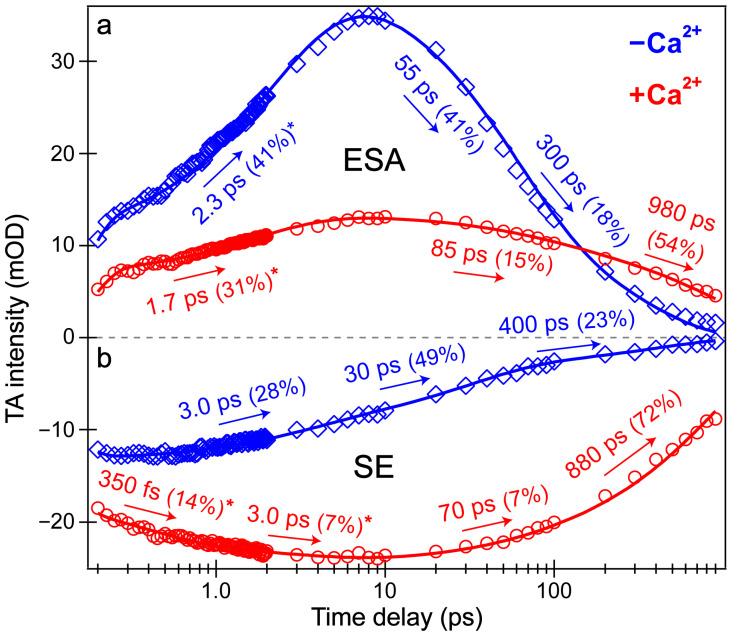
Probe-dependent fs-TA signal intensity dynamics of the (**a**) ESA and (**b**) SE features following 400 nm excitation of the Ca^2+^-free (blue diamonds) and Ca^2+^-bound (red circles) REX-GECO1 biosensor. Individual data points are overlaid with the least-squares multi-exponential fits (solid curves, color-coded), the retrieved time constants and weighted amplitudes are listed accordingly. The spectral integration regions for the Ca^2+^-free biosensor’s ESA and SE bands are 520–532 and 581–604 nm, respectively. The spectral integration regions for the Ca^2+^-bound biosensor’s ESA and SE are 523–535 and 583–620 nm, respectively. The asterisks by certain amplitude percentages indicate the intensity rise components from the best fits.

**Figure 5:**
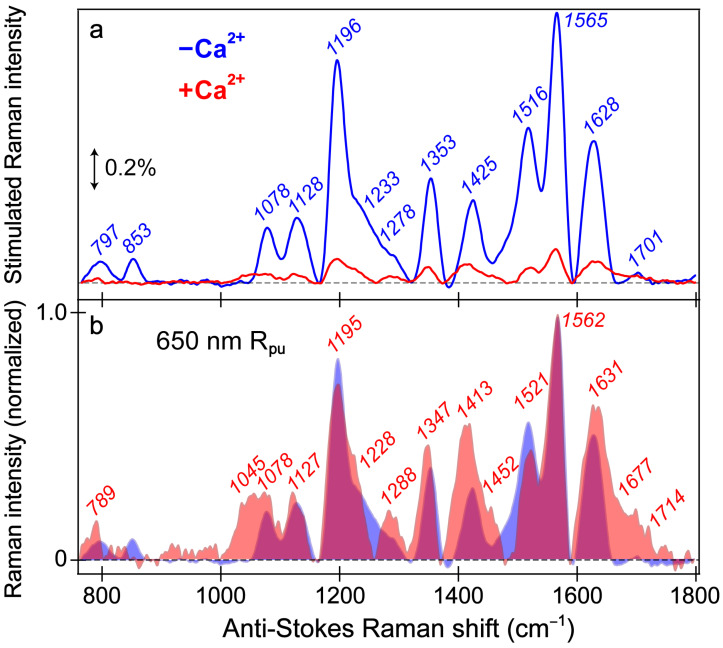
Ground-state FSRS spectra of REX-GECO1 without (blue) and with (red) calcium ions (Ca^2+^) in pH 7 buffer. (**a**) FSRS spectra recorded with a 650 nm Raman pump (R_pu_) and a bluer Raman probe on the anti-Stokes side. The double-arrowed line represents the stimulated Raman intensity magnitude of 0.2%. The stimulated Raman shift axis is multiplied by −1 to show positive vibrational frequencies. (**b**) The normalized FSRS spectra enhance peak comparisons with the magenta shade highlighting the overlapped Raman peaks of the Ca^2+^-free (blue shade) and Ca^2+^-bound (red shade) biosensors. The peak center frequencies (color-coded) from least-squares gaussian fits are listed for both biosensor states.

**Figure 6:**
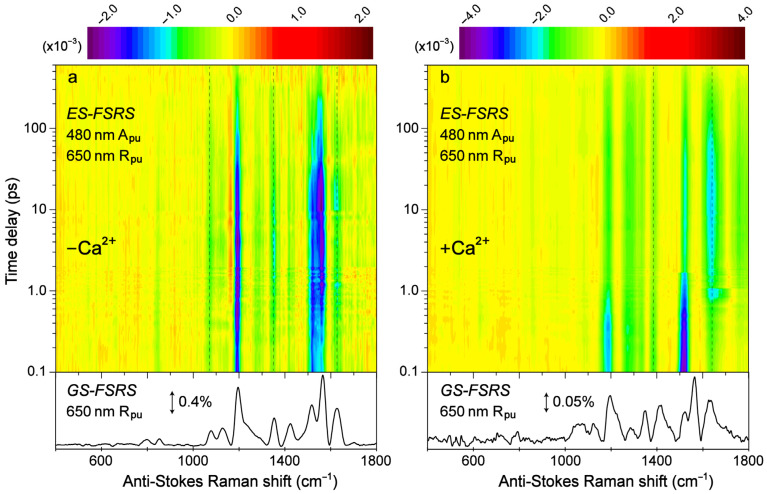
Contour plots of the excited-state (ES)-FSRS spectra of the (**a**) Ca^2+^-free and (**b**) Ca^2+^-bound REX-GECO1 biosensor in pH 7 aqueous buffer solutions. The FSRS data were collected with a 480 nm actinic pump (A_pu_), 650 nm Raman pump (R_pu_), and a bluer Raman probe on the anti-Stokes side. The color-coded stimulated Raman intensities (×10^−3^) are indicated by the color bar above each contour plot. The ground-state (GS)-FSRS spectra (without 480 nm A_pu_) are shown below each contour plot, with the denoted stimulated Raman intensity magnitude of 0.4 and 0.05% for the Ca^2+^-free and -bound biosensors, respectively (due to different resonance conditions achieved experimentally). The stimulated Raman shift axis is both multiplied by −1 for the GS- and ES-FSRS spectra. The vertical dashed lines highlight several key transient vibrational peaks analyzed below.

**Figure 7:**
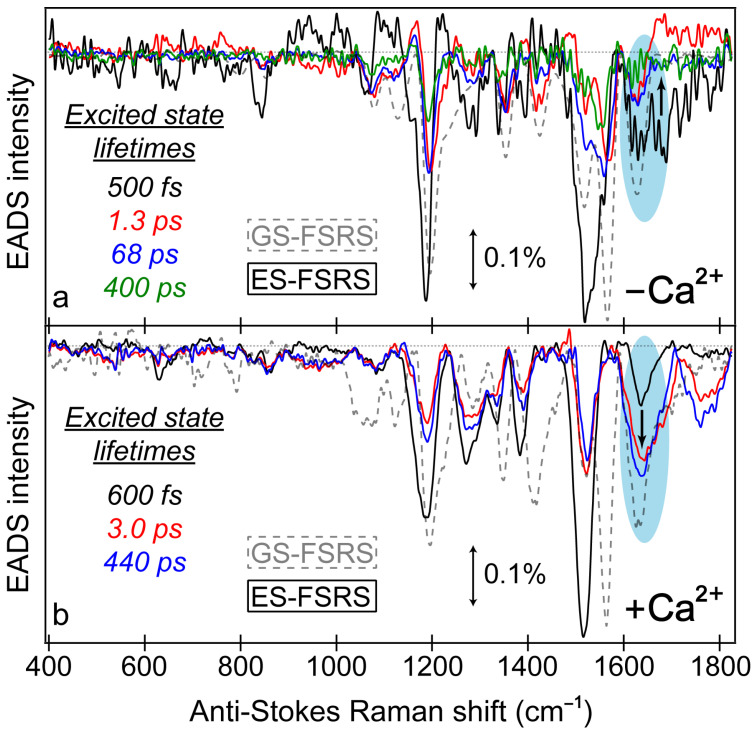
Global analysis of the excited-state FSRS spectra of REX-GECO1 biosensor in the (**a**) Ca^2+^-free and (**b**) Ca^2+^-bound state after 480 nm excitation with a 650 nm Raman pump (in pH 7 buffer), collected on the anti-Stokes side with a bluer Raman probe. The progression of the excited-state Raman peaks is visualized by the sequential evolution-associated difference spectra (EADS, see black→red→blue→green traces) with color-coded lifetimes (listed in the insets). Transient excited-state peaks (color-coded solid traces) are overlaid with the ground-state spectra (gray dashed traces). The double-sided arrows depict a stimulated Raman intensity of 0.1%. The cyan shaded ellipses highlight the contrasting vibrational peak dynamics above 1600 cm^−1^ between the Ca^2+^-free and bound biosensors, marked by the black bolded arrow in each panel.

**Figure 8:**
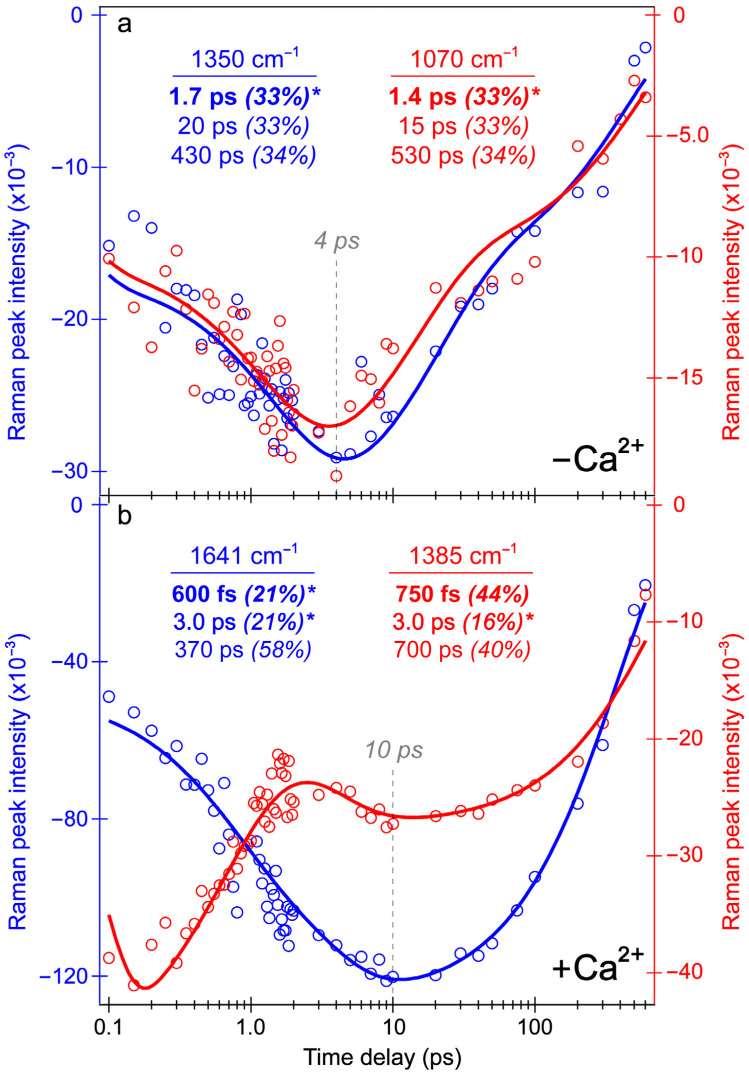
Integrated peak intensity dynamics from the REX-GECO1 biosensor’s excited-state FSRS spectra in pH 7 aqueous buffer. Intensity dynamics of the Raman bands centered at (**a**) 1350 cm^−1^ (blue) and 1070 cm^−1^ (red) of the Ca^2+^-free biosensor, and (**b**) 1641 cm^−1^ (blue) and 1385 cm^−1^ (red) of the Ca^2+^-bound biosensor. The data points (color-coded circles) are overlaid with the least-squares fits (solid curves) on a logarithmic timescale (horizontal axis). The retrieved time constants and amplitude weight percentages are listed accordingly, with the asterisks denoting the intensity rise components. Gray dashed lines highlight distinct delay times for the SE bands to reach maximal intensity magnitudes during the excited-state spectral evolution of the Ca^2+^-free (~4 ps in (**a**)) and bound (~10 ps in (**b**)) biosensors.

Intriguingly, the blue shoulder of the Ca^2+^-free ESA band (~490–510 nm, see Figure 3b) rises much less than the main positive peak to the red side (~510–540 nm). For an in-depth analysis, the probe-dependent slices of various TA features were taken and fit with exponential functions (Figure 4), which in large part reflect the global analysis results with a few additional insights. The mirror-like dynamics of the Ca^2+^-bound biosensor’s ESA and SE bands (red traces in Figure 4a,b) infer that they track the same electronic excited state. Notably, the 1.7 ps rise of the ESA is reminiscent of the biexponential rise of the SE feature (350 fs and 3 ps) with a weighted average of ~1.2 ps. This result infers that an inhomogeneous protonated population can undergo multiple ESPT pathways with varying time constants: <140 fs, 350 fs, and 3 ps. The sub-140 fs pathway is necessary due to the unambiguous and prompt rise of the I* SE immediately following time zero (Figure 2a and Figure 3b). The Ca^2+^-bound SE is significantly more intense (~18 mOD magnitude) near time zero versus the Ca^2+^-free case (~12 mOD), providing additional support for this ultrafast ESPT pathway because the Ca^2+^-bound B form population is greatly reduced compared to the Ca^2+^-free B form population at equilibrium (Figure 1b,c). A prior report on REX-GECO1 noted this chromophore inhomogeneity in the Ca^2+^-bound biosensor [18], this notable conformational diversity can be further evaluated by the ground- and excited-state FSRS structural characterization (see Figure 5, Figure 6, Figure 7 and Figure 8 in Section 3.3 below). The other probe-dependent dynamics (70–85 ps and 880–980 ps components) occur with similarly weighted amplitudes and are largely reflective of the global analysis results (Figure 3d).

In sharp contrast, the initial dynamics of the Ca^2+^-free biosensor are noticeably different, clearly breaking the mirror-like pattern observed for the Ca^2+^-bound biosensor. The B* (major) and I* (minor) SE decays with an ~3 ps time constant while the ESA rises significantly with a 2.3 ps time constant, confirming that the SE and ESA features report on different electronic excited states. The overall correlated rise/decay dynamics of the ESA/SE bands suggest that the twisting of the B* form could contribute to the dark-state formation, signified by the rise of ESA. As discussed above, the ~3 ps SE decay tracks the depletion of the B*/I* planar fluorescent state while the concurrent ESA rise reports on the dark-state formation via the chromophore twisting motions. The subsequent dynamics of the Ca^2+^-free biosensor on tens to hundreds of ps timescales are similar to global analysis with largely matching weighted amplitudes (Figure 3b). Upon comparing the intermediate decay of the excited-state features, the Ca^2+^-bound biosensor decays much less (7–15%, see red traces in Figure 4) than the Ca^2+^-free counterpart (41–49%, see blue traces in Figure 4), corroborating that the Ca^2+^-free biosensor possesses a more flexible chromophore.

Several additional probe-dependent slices of the fs-TA spectra were taken and compared to substantiate the retrieved dynamics (Figure S2). The Ca^2+^-bound biosensor dynamics reveal a 300 fs decay of the A form GSB band, tracking the initial recovery of the A form population and matching the ultrafast ESPT time constant of 350 fs (Figure 4b, red trace). Upon first glance, this timescale should reflect the A*→I* transition, not the A*→A transition. However, the ESPT step does indirectly lead to the recovery of the original A form via A*→I*→hot B and/or hot A→A, so the proton transfer step should be reflected in the overall A form recovery dynamics due to the correlated rate constants in kinetic modeling [53]. The GSB recovery continues to proceed with ~30 and 700 ps time constants, reflecting the nonradiative return of the excited-state population to the ground state during ultrafast conformational motions and a mixture of radiative/nonradiative transitions encapsulated by the apparent fluorescence lifetime, respectively. Based on the kinetic analysis of the fs-TA spectral data via both probe-dependent intensity dynamics and global analysis, the following kinetic scheme has been developed to explain the ultrafast light-induced response of the Ca^2+^-bound biosensor:

A→A*→I*→B′/A′→A

where A, A*, and A′ represent the electronic ground, excited, and hot ground state of the protonated chromophore, respectively. I* represents the intermediate electronic excited state after proton transfer in an unrelaxed protein environment, and B′ represents the hot electronic ground state of the deprotonated chromophore. The A* is formed immediately following photoexcitation, which quickly converts to I* with several time constants including <140 fs (within the cross-correlation time of the instrument), ~350 fs, and 3.0 ps (Figure 3d and Figure 4). The apparent fluorescence lifetime of I* approaches 1 ns, which represents an average measure of how long I* remains in the excited state [38,43] before reaching the hot ground state of the protonated and/or deprotonated chromophore, eventually returning to the original ground state, A. The limited time window of the fs-TA setup (see Section 2.2 above) does not allow for tracking of the hot ground state species and could be the focus of a future investigation.

Last but not least, a bluer slice of the ESA feature was taken for the Ca^2+^-free biosensor spectra to compare to the redder slice of the same ESA. The time constants from the intensity dynamics of the bluer slice (Figure S2, 501 ± 8 nm) are 2.0, 40, and 270 ps, quite similar to the time constants needed to fit the redder slice (Figure 4a, 526 ± 6 nm): 2.3, 55, and 300 ps. However, the weighted amplitudes are significantly different for the ~2 ps rise time constant: the bluer ESA rises much less (26%) than the redder ESA (41%), also apparent in the global analysis spectra (Figure 3b, red→blue trace). We surmise that the reduced rise magnitude of the bluer ESA is owing to spectral overlap with the adjacent A form GSB to the blue side (Figure 3b). Upon integrating this GSB feature and inspecting the peak intensity dynamics (see Figure S2, 442 ± 12 nm), the A form GSB also rises with a similar 2.2 ps time constant. In essence, a rising negative (GSB) feature overlapped with a rising positive (ESA) feature can decrease the apparent rise of both features. Notably, the similar dynamics of the blue/red ESA and GSB features (Figure 4a and Figure S2) imply that the spectral overlap does not significantly affect the retrieved time constants; however, the weighted amplitudes are subject to such contamination (especially for the bluer ESA band that is more overlapped with the adjacent GSB band) and thus require caution for in-depth interpretations. Similarly, the following kinetic scheme for the Ca^2+^-free biosensor has been rigorously developed from fs-TA data according to the ultrafast spectral evolution of the electronic features and subsequent analysis:

A→A*→I1*→A′→A and B→B*→I1*→B′→B

where A, A*, and A′ again represent the electronic ground, excited, and hot ground state of the protonated chromophore, while B, B*, and B′ represent the electronic ground, excited, and hot ground state of the deprotonated chromophore, respectively. Notably, I1* represents the dark-state intermediate of both the protonated and deprotonated chromophores. Similar to the aforementioned Ca^2+^-bound case, the A* and B* states of the Ca^2+^-free biosensor are formed immediately following photoexcitation. The protonated and deprotonated chromophore excited states rapidly reach the dark state, I1*, with an average time constant of 2–3 ps before I1* returns to the electronic ground state via nonradiative relaxation with a ~300–400 ps time constant (Figure 3 and Figure 4).

### 3.3. Equilibrium Structural Characterization of REX-GECO1 with and without Calcium Ions Helps to Rationalize Distinct Excited-State Vibrational Signatures via Femtosecond Stimulated Raman

To structurally characterize the differences and similarities between the chromophore (CRO) of the REX-GECO1 biosensor with and without calcium ions, the ground-state (GS)-FSRS measurements were performed in neutral pH aqueous buffer solution (Figure 5). This highly sensitive and versatile table-top structural dynamics technique [35,37,38,40,44,57,80] should reveal differences between the more compressed cavity volume of the Ca^2+^-bound biosensor with a stronger CRO-Glu80 interaction compared to the more spacious local environment of the Ca^2+^-free biosensor with a much reduced polar interaction due to the more distant Glu80. The Raman pump (R_pu_) wavelength of 650 nm can largely be considered off-resonance (far from the ground state absorption band, see Figure S1a) [81], although the R_pu_ is much more resonant with the deprotonated chromophore (B form) than the protonated chromophore (A form), especially for the Ca^2+^-free biosensor with an appreciable B population absorbing at 581 nm in pH 7 buffer (Figure 1b). As a result, the stimulated Raman peaks of the Ca^2+^-free biosensor are much more intense (7–10 fold enhancement) than the Ca^2+^-bound counterpart (Figure 5a).

The similarity of vibrational peaks across a broad spectral window upon a comparison between the Ca^2+^-free and -bound biosensors provides useful insights into the chromophore structure. While these GS-FSRS peaks mainly originate from the deprotonated chromophore in the Ca^2+^-free case given the large B form population and enhanced resonance contributions, this assignment is less clear in the Ca^2+^-bound case because there is a dominant A population (481 nm absorption peak) at neutral pH yet the smaller B population (560 nm) achieves significantly better resonance (Figure S1a). While there is likely a nonnegligible contribution from the protonated chromophore, we consider the Ca^2+^-bound state spectrum to be largely representative of the B form based on similarities of the peak frequencies and patterns shared with the Ca^2+^-free state spectrum (Figure 5a). The normalized spectra (Figure 5b) allow for a better comparison between the Ca^2+^-free and -bound states, which feature a notable Raman peak overlap (magenta shade) and support a common spectral origin from the deprotonated chromophore. An even more off-resonance Raman pump at 800 nm was employed to provide further verification (Figure S3), wherein the observed Raman peak pattern differs significantly from that with the bluer 650 nm R_pu_. There is appreciably more protonated chromophore (A form) absorption near 400 nm in the Ca^2+^-free state than the Ca^2+^-bound state in pH 7 buffer (Figure 1b,c), leading to the observed stronger Raman peaks in the Ca^2+^-free biosensor (Figure S3). Therefore, the Raman spectral differences in Figure 5a versus Figure S3 are likely because the 800 nm R_pu_ resonantly enhances the A form via two-photon absorption [82,83]. Minor contributions from the deprotonated chromophore are expected given the more optimal resonance conditions with a red-wavelength R_pu_, especially for the Ca^2+^-free biosensor with a large B form population. Moreover, the larger electric polarizability of the chromophore [38,79] in a more flexible environment in the Ca^2+^-free biosensor is in accord with the generally much larger Raman peak intensities than the Ca^2+^-bound counterpart implementing both R_pu_ wavelengths (Figure 5 and Figure S3).

The overall similarity of the vibrational spectra with and without calcium is somewhat expected given both FP-based biosensors share the identical chromophore chemical structure responsible for these peaks. Thus, differences in the peak shape, intensity patterns, and frequencies give sensitive information regarding the different interactions the chromophore experiences with the local environment in the presence and absence of calcium ions (i.e., binding at the adjacent CaM subunit). While the local environment is expected to be different for the Ca^2+^-free/-bound biosensors without/with the Glu80 residue nearby, this weakened/strengthened polar interaction between Glu80 and the chromophore should not play as large a role for the deprotonated chromophore due to repulsion between the negatively charged chromophore and Glu80 in neutral pH buffer. Hence, even though the Ca^2+^-bound biosensor has a more compressed cavity with a more proximal Glu80, the differences between the Ca^2+^-free and -bound biosensor spectra are diminished. The GS-FSRS with an 800 nm R_pu_ (Figure S3) confirms this point, wherein the vibrational spectra largely representing the protonated (A form) chromophores manifest some discernible differences between the Ca^2+^-free and -bound biosensors because the protonated chromophore of the latter biosensor establishes a stronger polar interaction with the adjacent negatively charged Glu80 residue [18,27].

The vibrational motions that act as sensitive probes reveal several insights pertaining to the CRO-local environment interactions. In general, the Ca^2+^-bound peaks are broader than the Ca^2+^-free counterparts; for example, the spectral regions from ~1000–1150, 1380–1500, and 1600–1750 cm^−1^ (Figure 5b), which are highlighted by the magenta shades that showcase the generally broader Raman peaks in the Ca^2+^-bound biosensor (red shade) than its Ca^2+^-free counterpart (blue shade). The increased broadness may arise from a more prominent contribution from the A form population upon Ca^2+^ binding; however, this observation also hints at the increased inhomogeneity of the chromophore in the Ca^2+^-bound biosensor. Of particular importance is the peak(s) above 1600 cm^−1^ assigned to the C=O stretch on the phenolate (P)-ring of the chromophore. First, the presence of this peak alone indicates that the spectra largely represent the B form since the C–OH stretch should be dominant in the A form at a different frequency. Second, the C=O stretching motion responsible for this vibrational peak is highly sensitive to interactions between the phenolate moiety and nearby Glu80 residue (Figure 1a). The notable broadness of this peak, especially compared to the Ca^2+^-free counterpart, implies the enhanced inhomogeneity of the Ca^2+^-bound biosensor’s chromophore in a more compressed environment since the chromophore may be forced into various conformations (i.e., with varied vibrational mode frequencies) that cannot interchange efficiently [82,84]. In contrast, the more spacious local environment inside the Ca^2+^-free biosensor allows the population to search phase space and find one dominant conformation intrinsic to the chromophore. This point is analogous to the narrow linewidth of a chromophore in the gas phase or dilute solution versus an amorphous thin film with a more heterogeneous distribution [85,86]. The inferred chromophore inhomogeneity from the broadness of the peaks, especially above 1600 cm^−1^, manifests itself in the fs-TA (see Section 3.2 above) and excited-state FSRS spectra tracking the various ESPT timescales in the Ca^2+^-bound biosensor (see below).

Transient structural dynamics of the Ca^2+^-free and -bound REX-GECO1 biosensor were observed via ES-FSRS upon excitation of the A form with a 480 nm actinic pump (Figure 6). The FSRS spectra were collected with a 650 nm Raman pump and a bluer white-light Raman probe on the anti-Stokes side (see Section 2.2 above), achieving dynamic resonance enhancement [37,38,43] with the SE band of the B*/I* state. Therefore, the ES-FSRS contour plots (Figure 6) represent an intriguing experimental condition wherein the protonated chromophore is excited while the structural motions of predominantly the deprotonated chromophore are observed, allowing for a comparative analysis of the ESPT (or lack of ESPT) reaction in real time [41,82]. There is a multitude of excited-state negative peaks that intriguingly evolve as the Ca^2+^-free (Figure 6a) and Ca^2+^-bound (Figure 6b) biosensor chromophore populations navigate the potential energy surfaces. Notably, to substantiate the ES-FSRS data quality, the excited-state stimulated Raman peaks are displayed lying on top of a broad spectral baseline (Figure S4), which essentially mimics the SE band from the fs-TA spectra (Figure 3b,d) for the independently measured Ca^2+^-free and -bound REX-GECO1 biosensors (Figure S4a,b). The spline-like baselines shown (black traces in Figure S4) can then be subtracted from the raw experimental data, resulting in “clean” and readily analyzable excited-state peaks with nearly flat baselines for the Ca^2+^-free and -bound biosensors (Figure S5) [34,37,38]. There are several shared excited-state peaks regardless of Ca^2+^ binding at ~1195, 1280, and 1520 cm^−1^. Furthermore, there are several excited-state peaks that are slightly shifted between the Ca^2+^-free (1350, 1628 cm^−1^) and Ca^2+^-bound (1332, 1385, and 1641 cm^−1^) biosensors. Interestingly, there are two additional ES-FSRS peaks at ~1070 and 1560 cm^−1^ in the Ca^2+^-free case (most prominent after ~5 ps in Figure S5, see the frequency labels therein) that are not observed upon excitation of the Ca^2+^-bound biosensor.

Similar to the detailed examination of the fs-TA spectra (Figure 3), global analysis of the ES-FSRS spectra (Figure 7) can help to reveal the underlying processes guiding the spectral evolution [53,87]. In the Ca^2+^-free and -bound biosensors, transient Raman peaks were systematically fit with the least amount of temporal components to achieve a satisfactory fit. In general, the Ca^2+^-bound peaks are more intense with a higher signal-to-noise ratio because the 650 nm R_pu_ achieves better resonance enhancement with the more intense deprotonated form SE in the Ca^2+^-bound biosensor than the Ca^2+^-free counterpart (Figure 3). Similarly, the 650 nm R_pu_ is more resonant with the ground-state absorption from the deprotonated chromophore without calcium ions (green trace in Figure S1a), thus leading to an increased GSB contribution and less spectral separation between the ground state (gray dashed traces) and excited state (solid traces) peaks for the Ca^2+^-free biosensor (Figure 7a). We remark that the ES-FSRS data trace at each time delay point has the GS-FSRS data trace subtracted, hence the apparent peak shift and intensity pattern change between the gray dashed and black solid traces in Figure 7 is reflective of the light-induced electron density redistribution across the chromophore ring system at an early time and the subsequent correlated conformational and electronic motions [38,72,74].

Notably, a minimum number of three temporal components was required to fit the Ca^2+^-bound biosensor’s ES-FSRS spectra with lifetimes of ~600 fs, 3 ps, and 440 ps (Figure 7b). Upon comparison to the fs-TA global analysis (Figure 3d) and probe-dependent intensity dynamics (Figure 4) in the Ca^2+^-bound state, the 70–85 ps component seems to be absent in the ES-FSRS global analysis results. In Figure 4, the pertinent SE feature’s intensity decay time constant has a minor weight (70 ps at 7%), it is thus reasonable that the pre-resonantly enhanced excited-state Raman peaks may not track this component with sufficient distinction to the other dynamic processes [35,38]. The reduction of the apparent fluorescence lifetime from ES-FSRS (440 ps) versus the relevant time constant from fs-TA (880 ps–1.1 ns) is likely due to some contributions from the aforementioned 70–85 ps component, although the reduced signal-to-noise ratio of ES-FSRS spectra could also play a role leading to an apparently reduced lifetime on the sub-ns timescale. Most importantly, the sequential EADS of the Ca^2+^-bound biosensor manifests a biphasic rise of the ~1641 cm^−1^ peak with characteristic lifetimes of 600 fs (black→red trace) and 3 ps (red→blue trace) in Figure 7b. This peak mainly assigned to the P-ring C=O stretch proves the multi-step ESPT pathways because such a peak would not be expected for the protonated chromophore (–COH) and it also rises with the same ESPT time constants corresponding to the I* SE band rise (see fs-TA results in Figure 3d and Figure 4b).

The ultrafast ESPT pathway within the cross-correlation time of our laser optical setup is further supported by the clear presence of the ~1641 cm^−1^ peak from time zero (Figure 7b, black trace). Upon 480 nm excitation of the Ca^2+^-bound biosensor with a dominant A form population, such a prominent peak would not be expected from time zero without a sub-140 fs ESPT step, although direct excitation of the diminished deprotonated population could contribute to some extent. The excited-state peak is close in frequency to the ground-state counterpart, which can also be assigned to the C=O stretch of the deprotonated chromophore given the resonance conditions (Figure 5). However, the ES Raman peak (1641 cm^−1^) is blue-shifted from the GS counterpart (1631 cm^−1^), likely due to a combination of factors including the altered electronic distributions and conformational states of the chromophores with various local environment interactions (e.g., H-bonding) for the populations in the anharmonic electronic ground and excited states [73,88,89,90]. This multi-step ESPT is in accord with the aforementioned inhomogeneity of the equilibrium protonated chromophore (A form) population due to the compressed cavity in the Ca^2+^-bound biosensor, with experimental evidence provided by the GS-FSRS peak width comparison across a broad spectral window (Figure 5b).

A majority of the Ca^2+^-bound biosensor’s excited-state peaks below 1600 cm^−1^ show notably different dynamics, including peaks centered around 1185, 1277, 1385, and 1515 cm^−1^ (Figure 7b), which initially decay with a 600 fs time constant (black→red trace), then rise with a 3 ps time constant (red→blue trace). This specific pattern can be better reflected by the intensity dynamics of the 1385 cm^−1^ mode assigned to the hydrogen (H)-motions throughout the chromophore (Table S1), which initially decays with a 750 fs time constant (44% weight) before a minor rise with a 3 ps time constant (16% weight, see Figure 8b, red trace). The dynamics of the other lower-frequency modes are all similar to the 1385 cm^−1^ mode (Figure 7b). These peak dynamics are contrasted by the intensity dynamics of the P-ring C=O stretching motion (Figure 8b, blue trace), which rise with equally-weighted time constants of 600 fs and 3 ps both attributable to ESPT. This key spectral signature can be clearly visualized by the baseline-subtracted spectra at several representative time delay points in Figure S5 (see the vertical green dashed lines), wherein the initial rise of the 1641 cm^−1^ mode is contrasted by the initial decay of the redder peaks (e.g., 1385 cm^−1^) following 480 nm excitation.

According to these clearly contrasting peak dynamics, we can assign the 1641 cm^−1^ mode to a “pure” photoproduct state (see Figure 8b for the characteristic mode intensity fit), because the chromophore P-ring C=O stretch (see Figure S6a and Table S1 for the related ground-state calculation-aided mode assignment, and Figure S6b for the corresponding normal mode illustration) is only expected to occur at this frequency post-ESPT. On the other hand, the 1385 cm^−1^ mode is a shared reactant-product mode given its associated H-motions throughout the chromophore that is insensitive to the protonation state (see Table S1 for the corresponding normal mode assignment). The mode intensity decay with a 750 fs time constant thus still represents the A*→I* transition, likely mixed with some Franck–Condon dynamics as the A* population descends down the steep potential energy surface following photoexcitation while the I* population starts to accumulate on the sub-ps timescale [38,56,57]. The intermediate rise on the 3 ps timescale occurs with a similar weight (16%) to the 3 ps rise of the 1641 cm^−1^ mode (21%), corroborating the shared reactant-product nature of this mode (Figure 8). Notably, the sub-ps decay of these lower-frequency modes in the Ca^2+^-bound biosensor, exemplified by the 1385 cm^−1^ peak, differs from the SE dynamics (Figure 4b) with a 300 fs rise time constant. According to these differing electronic and vibrational intensity dynamics, the spectral evolution of the ES-FSRS peaks below ~1600 cm^−1^ must primarily reflect the intrinsic electric polarizability and population change during ESPT [37], which is consistent with their more delocalized normal mode nuclear motions (i.e., beyond the phenolate C=O end and/or P-ring), so the observed Raman peak intensity dynamics cannot be explained by the change of resonance enhancement factor alone [38,91].

For comparison, the Ca^2+^-free biosensor’s ES-FSRS intensity dynamics of the 1070 and 1350 cm^−1^ modes (Figure 8a) are representative of several modes with an initial rise time constant of 1.4–1.7 ps (Figure 8a). Similar to a majority of the Ca^2+^-bound biosensor peaks below 1600 cm^−1^, the intensity dynamics of these Ca^2+^-free biosensor peaks also differ significantly from the B*/I* state SE dynamics (see Figure 3b and Figure 4b), which initially decays on the 2–3 ps timescale. Although the 650 nm Raman pump is pre-resonant with the SE feature assigned to a planar B* fluorescent state, these peaks seem to track the dark-state formation upon twisting of the flexible chromophore in the Ca^2+^-free biosensor. Notably, the rise of these Raman modes nicely matches the rise of the ESA band (Figure 3b and Figure 4a) assigned to a dark state with contributions from the protonated (major) and deprotonated (minor) chromophores on the ~2 ps timescale. Given the resonance conditions (Figure S1a), the similarity between ES- and GS-FSRS peaks of the B form (Figure 7a), and a dominant B form population of the Ca^2+^-free biosensor in neutral buffer (Figure 1b), these ES-FSRS peaks can be assigned to a transition from the planar B* fluorescent state to the twisted conformation that is a dark state signature. Both the 1070 and 1350 cm^−1^ peaks primarily involve the chromophore P-ring and bridge H-motions (see Figure S6c for an illustration of the calculated normal mode at ~1362 cm^−1^, also highlighted in Table S2). The rise of these peaks contradicts the apparent decay of the planar B*/I* state SE band and worsening resonance condition (see Figure 3b), while a growing ESA band (475–550 nm) is visibly distant from the Raman pump (650 nm). Therefore, the observed Raman mode intensity rise must reflect the intrinsic electric polarizability increase upon twisting from the coplanar chromophore.

Interestingly, the rise of a similar mode assigned to H-motions centered on the P-ring has been observed for another fluorescent protein termed the least evolved ancestor (LEA) as the deprotonated chromophore twists from planarity [45,79]. Both deprotonated chromophores within LEA and the Ca^2+^-free REX-GECO1 biosensor exist in a relatively spacious protein pocket; however, the chromophore still experiences various short- and long-range interactions with nearby protein residues that affect the chromophore properties. We reckon that the chromophore twisting motion disrupts the interactions with the local environment, thus leading to a notable increase of the electric polarizability pertaining to characteristic vibrational motions involving the hydrogens on the P-ring with an altered electronic configuration (i.e., the P- and I-ring conjugation through the methine bridge is clearly affected [92,93,94]). Such an experimental finding warrants high-level calculations of the REX-GECO1 biosensor’s MYG chromophore, potentially involving quantum mechanics—molecular mechanics (QM/MM) simulations that factor in the protein local environment [58,95,96,97]. Notably, our prior quantum calculations on the LEA chromophore revealed a significantly increased Raman activity of the 1340 cm^−1^ peak involving P-ring H-motions as the P-ring twists away from planarity toward a two-ring perpendicular configuration [45].

Notably, not all of the Ca^2+^-free ES-FSRS peaks display the same dynamics as the 1070 and 1350 cm^−1^ modes that are considered marker bands for the dark-state formation. In Figure 7a, the 1628 cm^−1^ peak does not initially rise like the aforementioned peaks; instead, the peak decays starting from time zero. The “pure” decay dynamics with ~500 fs, 1.3 ps, 68 ps, and 400 ps lifetimes (Figure 7a) largely match the decay dynamics of the B* state SE band with ~2, 44, and 330 ps time constants (Figure 3b), so the 1628 cm^−1^ peak mostly tracks the nonradiative transition of the deprotonated chromophore (B*→B). The presence of this P-ring C=O/C=C stretch peak (Table S2) from time zero again provides support for direct excitation of the B form by 480 nm light. Ultrafast ESPT within the cross-correlation time of the optical setup remains a possibility as we mentioned, yet it remains unclear how such an efficient ESPT would proceed with a flexible chromophore in a spacious protein pocket with no adjacent proton acceptors (see Figure 1a for the Ca^2+^-bound biosensor as a contrast). The chromophore inhomogeneity provides a plausible explanation that a minor subpopulation could proceed ultrafast ESPT, yet the main subpopulation would undergo the chromophore-ring twists to reach a dark state [98,99]. Perhaps more importantly, the lack of an ultrafast rise of the 1628 cm^−1^ peak following time zero proves that ESPT does not occur significantly within the Ca^2+^-free biosensor. This finding is apparent by comparing the Ca^2+^-free and -bound baseline-subtracted ES-FSRS traces (see Figure S5a–e), where the latter biosensor shows a pronounced, intense rise of the 1641 cm^−1^ peak that is juxtaposed by the rather “mundane” decay dynamics of the 1628 cm^−1^ peak of the chromophore in the Ca^2+^-free biosensor.

### 3.4. Rational Design Principles from Spectroscopic Insights to Improve the Performance of REX-GECO1 Biosensor

To further improve the red/green emission ratio change, several general principles can be applied to rationally design the chromophore and local environment within the REX-GECO1 biosensor. In order to achieve the largest ratiometric enhancement while bound to calcium ions, the red emission intensity needs to be increased while reducing the green emission. The solution is relatively simple in theory, but difficult to achieve practically: improving the ESPT upon photoexcitation of the protonated chromophore that can lead to more red B* emission and less green A* emission [72,100,101,102,103]. There are primarily two means to achieve this goal: modifying the local environment and the chromophore [90,101,103,104]. With regard to the first proposition, the local environment could be altered to shorten the distance between Glu80 and the P-ring of the chromophore. If this distance could be shortened from 3.0 Å (Figure 1a) to ~2.7 Å, the ESPT probability and rate would increase, leading to even less green emission and more red emission. Furthermore, other nearby residues could be modified to more bulky amino acids that increase polar interactions with the chromophore. This approach could not only funnel the various inhomogeneous populations toward ESPT instead of other futile relaxation pathways but also further conformationally restrain the chromophore, thus limiting its twisting-induced nonradiative transitions to improve the FQY, brightness, and dynamic range. It can be difficult to predict the exact changes in the electronic properties of the chromophore upon modification via the incorporation of non-canonical amino acids (ncAAs) [105,106]; however, the ESPT probability can be fine-tuned by adding electron-withdrawing groups to the P-ring of the chromophore [101,103]. The incorporation of other electron-withdrawing and/or -donating substituents throughout the chromophore framework could additionally red-shift the deprotonated emission band of the RFP-based chimeric biosensor with a larger Stokes shift, which is beneficial for *in vivo* measurements, owing to the deeper penetration depths of red and near-infrared light [107,108].

With regards to the Ca^2+^-free biosensor, the primary objectives are similar but opposite: reduce the emission from the red B*/I* form and increase the emission from the green A* chromophore. To improve the green emission of the Ca^2+^-free biosensor, the solution is fairly similar to the Ca^2+^-bound biosensor: confine the chromophore by modifying the local environment so that the twisting-induced dark-state formation becomes less feasible. This rational design principle could have unintended consequences though; for example, the dark-state formation may quench ESPT to a certain degree in the absence of calcium ions, and by reducing the capability for the chromophore to twist, the ESPT may become more prominent. Furthermore, the rational design principles applied to increase/decrease the red/green emission of the Ca^2+^-bound biosensor must have the opposite effect for the Ca^2+^-free biosensor to achieve the largest emission-ratiometric enhancement. Thus, by improving the ESPT propensity for the Ca^2+^-bound biosensor, the ESPT pathway may also become enhanced for the Ca^2+^-free biosensor, although the structural reorganization of the FP subunit upon CaM wrapping around the calcium ions and M13 peptide could affect the emission outcomes. Perhaps the large cavity space experienced by the chromophore in the Ca^2+^-free biosensor can render these changes negligible without calcium while having a more pronounced effect with calcium. The detrimental effects of this design strategy for the Ca^2+^-free biosensor may be outweighed by the positive effects for the Ca^2+^-bound biosensor. With little evidence for ESPT inside the Ca^2+^-free biosensor from this current investigation, the primary means to reduce the red emission upon 480 nm excitation of the Ca^2+^-free biosensor is to increase the spectral separation between the protonated and deprotonated forms’ absorption bands. The pernicious direct excitation of the deprotonated chromophore leads to undesirable red emission upon 480 nm excitation; therefore, by red-shifting the deprotonated absorption peak there will be less spectral overlap and hence a reduced red emission. The most effective means to improve this spectral separation may be through the incorporation of ncAAs by engineering strategic electron-donating and -withdrawing substituents throughout the chromophore framework [106,109,110,111].

Intrinsically, the interplay between the effects of direct excitation, ESPT capability, and dark-state formation can be challenging to predict by modifying the local environment surrounding the chromophore in addition to the chromophore itself. However, the aforementioned rational design principles aiming to enhance ESPT in the Ca^2+^-bound biosensor by reducing the CRO-Glu80 distance may aid the spectral separation between the A and B form absorption bands for the Ca^2+^-free biosensor. In particular, at neutral pH the shifted (likely moving closer) anionic Glu80 residue may further stabilize the ground-state protonated chromophore via hydrogen bonds while the interatomic charge repulsion may destabilize the ground state of the deprotonated chromophore, which could then blue-shift and red-shift the Ca^2+^-free biosensor’s A and B form absorption bands, respectively. These fundamental principles and interaction schemes may be more broadly applicable to other calcium biosensors in addition to various indicators for physiologically relevant ions [77].

## 4. Conclusions

In summary, we spectroscopically investigated a recently developed ratiometric calcium biosensor termed REX-GECO1 that is capable of achieving a red/green emission ratio change of ~300% that outperforms many other FRET and standalone FP-based calcium indicators. The steady-state spectra showcase a compressed cavity size of the Ca^2+^-bound biosensor with a proximal Glu80 residue, resulting in a p*K*_a_ change of the chromophore and enhanced ESPT that enables the ratiometric enhancement following excitation of the protonated chromophore. In tandem with steady-state electronic results, fs-TA spectroscopy reveals that the more flexible RFP chromophore within a spacious Ca^2+^-free biosensor pocket quickly reaches a dark state upon the chromophore twist with an ~2 ps time constant. In contrast, the Ca^2+^-bound biosensor undergoes a multi-step ESPT as the I* state SE feature rises with sub-140 fs, 350 fs, and 3 ps time constants due to an inhomogeneous distribution of equilibrium conformations, corroborated by GS-FSRS fingerprints. The most striking evidence for the prominent and greatly diminished ESPT with and without calcium ions, respectively, is conspicuous from the ES-FSRS spectra that are aided by global analysis. The biphasic rise of the 1641 cm^−1^ marker band assigned to the P-ring C=O/C=C stretch of the deprotonated chromophore matches the I* state formation time constants of ca. 350–600 fs and 3 ps in the Ca^2+^-bound REX-GECO1 biosensor. In contrast, several vibrational modes in the Ca^2+^-free biosensor’s ES-FSRS spectra rise with a characteristic dark-state formation time constant of 1.4–1.7 ps as the chromophore P-ring twists, leading to an electric polarizability increase for the observed ~1070 and 1350 cm^−1^ marker bands affiliated with hydrogen motions centered on the P-ring.

In essence, we have established in this work an effective feedback loop between the equilibrium properties retrieved from steady-state measurements and better explained these properties through time-resolved (ultrafast) spectroscopic techniques. One of the key advantages of this experimental strategy is that we are able to study dynamic processes on the intrinsic molecular timescales, thus allowing us to track the light-induced processes in real time. This approach has enabled us to bridge the gap between the microscopic processes and macroscopic properties that power REX-GECO1 as a sensitive biosensor. In addition, many spectroscopic techniques are noninvasive and well-suited for a direct translation to *in vivo* measurements. Finally, the synergistic use of fs-TA and FSRS can reveal intricate coupling between the electronic and structural (vibrational) manifolds. Based on these fundamental mechanistic insights into an FP-based biosensor in action, we proposed a series of executable strategies to rationally design such genetically encodable biosensors with improved properties for broader use in fluorescence-based bioimaging. With the ongoing dedicated and collaborative efforts between protein engineers, spectroscopists, biophysicists, bioimaging, and the life science community, we envision these proposed modifications can further improve the properties of REX-GECO1 and related biosensors by increasing the dynamic range and brightness to allow for more sensitive bioanalyte detection at even lower levels.

## Data Availability

All data needed to evaluate the conclusions in the paper are present in the paper and the Supplementary Materials.

## Supplementary Materials

The following supporting information [20,35,36,37,38,42,43,46,82,83] can be downloaded at: https://www.mdpi.com/article/10.3390/bios13020218/s1, Figure S1: Steady-state electronic spectroscopy of the Ca^2+^-free and -bound biosensors with the absorption and excitation-dependent emission spectra; Figure S2: Probe-dependent fs-TA data with least-squares fits of the Ca^2+^-free and -bound biosensors after 400 nm excitation in pH 7 buffer; Figure S3: GS-FSRS spectra of REX-GECO1 with an 800 nm Raman pump; Figure S4: ES-FSRS spectra of REX-GECO1 at representative time points with spline-like baselines; Figure S5: ES-FSRS spectra of REX-GECO1 at representative time points after the spectral baseline subtraction; Figure S6: Calculated Raman spectrum and experimental GS-FSRS with key normal modes of REX-GECO1 from quantum calculations; Table S1: Calculated ground state Raman and experimental FSRS peak frequencies with vibrational mode assignments of the deprotonated MYG chromophore in the Ca^2+^-bound biosensor; Table S2: Calculated ground state Raman and experimental FSRS peak frequencies with vibrational mode assignments of the deprotonated MYG chromophore in the Ca^2+^-free biosensor.
