# Supplementary material for: Delineating Ultrafast Structural Dynamics of a Green-Red Fluorescent Protein for Calcium Sensing
Source: Biosensors (Basel). 2023 Feb 2;13(2):218. doi: 10.3390/bios13020218 (PMC9954042; doi:10.3390/bios13020218)
Supplement: Supplementary file 1 [file biosensors-13-00218-s001.zip › biosensors-2126487-supplementary.pdf]

biosensors

Supporting Information

Delineating Ultrafast Structural Dynamics of a Green-Red Fluorescent Protein for Calcium Sensing

Taylor D. Krueger, Longteng Tang and Chong Fang *

Department of Chemistry, Oregon State University, 153 Gilbert Hall, Corvallis, OR 97331, USA
* Correspondence: chong.fang@oregonstate.edu; Tel.: +1-541-737-6704;
Web: https://fanglab.oregonstate.edu/

Table of Contents

1. Supplementary Figures

Figure S1. Steady-state electronic spectroscopy of the Ca^{2+}-free and -bound biosensors in aqueous buffer with the absorption and excitation-dependent emission spectra 2

Figure S2. Probe-dependent transient absorption (fs-TA) data with least-squares fits of the Ca^{2+}-free and -bound biosensors after 400 nm excitation in pH 7 buffer 3

Figure S3. Ground-state FSRS of REX-GECO1 with an 800 nm Raman pump 4

Figure S4. Excited-state FSRS of REX-GECO1 with broad spectral baselines 5

Figure S5. Excited-state FSRS of REX-GECO1 biosensors at representative time delay points after baseline subtraction in the Ca^{2+}-free versus -bound states 6

Figure S6. Calculated Raman spectrum and experimental ground-state FSRS with key normal modes of REX-GECO1 from quantum calculations 7

2. Supplementary Tables

Table S1. Calculated ground state Raman and experimental FSRS peak frequencies with mode assignments of the deprotonated chromophore in the Ca^{2+}-bound biosensor 8

Table S2. Calculated ground state Raman and experimental FSRS peak frequencies with mode assignments of the deprotonated chromophore in the Ca^{2+}-free biosensor 9

3. Supplementary References 10

1. Supplementary Figures

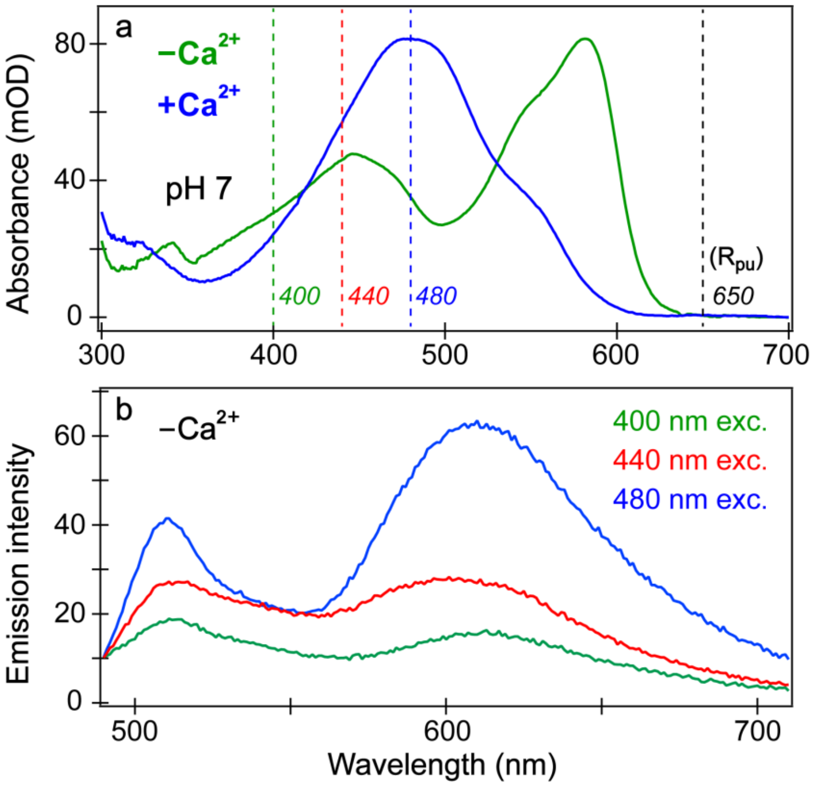

Figure S1. Steady-state electronic spectra of the REX-GECO1 biosensor in aqueous buffer. **(a)** Absorption spectra of the Ca^{2+}-free (green) and Ca^{2+}-bound (blue) biosensor in pH 7 buffer solutions. The green, red, and blue dashed lines indicate the 400, 440, and 480 nm excitation wavelengths, respectively, corresponding to the emission spectra in panel **b**. The black dashed line denotes the Raman pump (R_{pu}) position (650 nm) used for the ground- and excited-state FSRS measurements. **(b)** Emission spectra of the Ca^{2+}-free biosensor in pH 7 buffer solution upon 400 nm (green), 440 nm (red), and 480 nm (blue) excitation with the identical entrance and exit slit width conditions in the fluorometer. The reduced signal-to-noise ratio is due to the intrinsically low fluorescence quantum yield (hence the emission photon counts) of the Ca^{2+}-free biosensor.

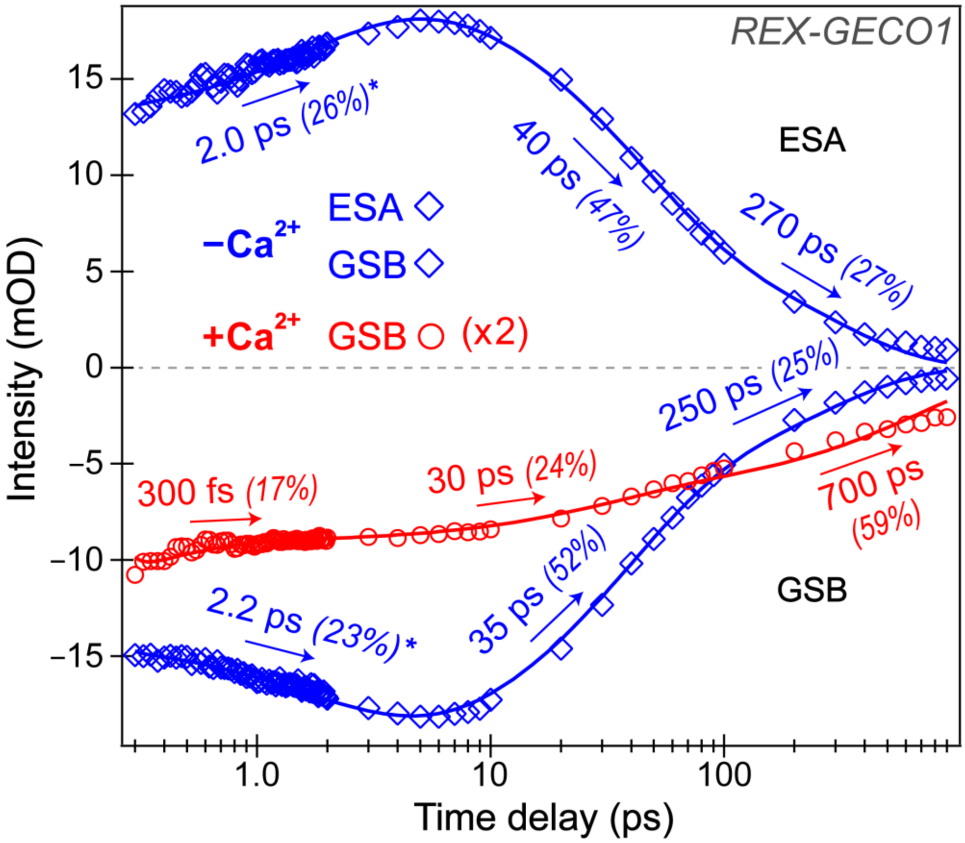

Figure S2. Probe-dependent fs-TA signal intensity dynamics of the Ca^{2+}-free (blue diamonds) and Ca^{2+}-bound (red circles) REX-GECO1 biosensor spectral features upon 400 nm excitation in pH 7 aqueous buffer solution with a logarithmic time axis. The individual data points are overlaid with the least-squares multiexponential fits (solid curves, color-coded), the retrieved time constants and weighted amplitudes are listed accordingly by the solid arrows representing the individual dynamic components. The spectral integration regions for the Ca^{2+}-free biosensor's ground-state bleaching (GSB, negative sign) and excited-state absorption (ESA, positive sign) bands are 430–454 and 493–509 nm, respectively. The spectral integration region of the Ca^{2+}-bound biosensor's GSB band is 437–460 nm. The asterisks by certain amplitude percentages indicate the intensity rise components from the best fits of TA data points.

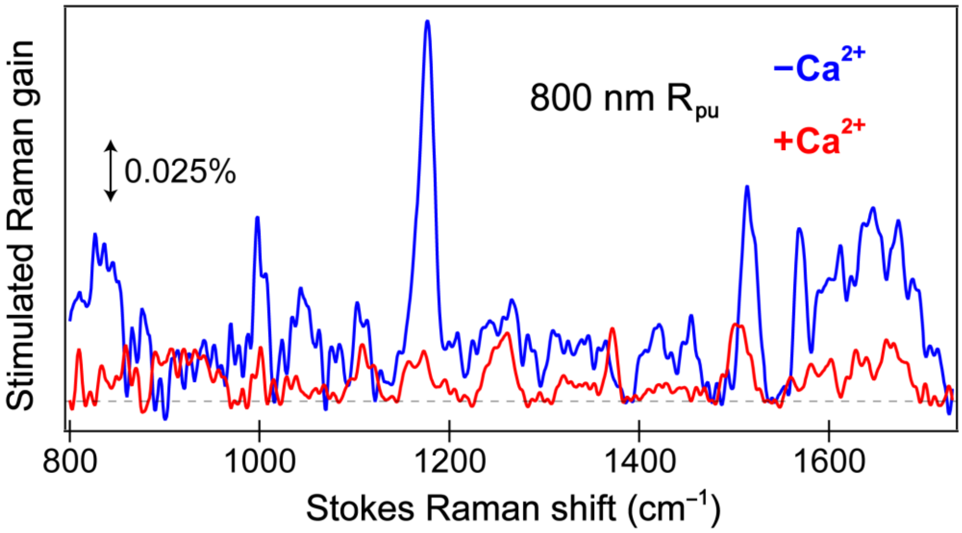

Figure S3. Ground-state FSRS spectra of the REX-GECO1 biosensor without (blue, –Ca^{2+}) and with (red, +Ca^{2+}) calcium ions in pH 7 buffer. The FSRS spectra were collected with an 800 nm Raman pump (R_{pu}) and a redder Raman probe on the Stokes side. The double-arrowed line denotes the stimulated Raman gain intensity magnitude of 0.025%, and the zero intensity is marked by the horizontal dashed line. The much stronger Raman peaks in the Ca^{2+}-free state (blue) are consistent with a higher absorption intensity at 400 nm in the Ca^{2+}-free biosensor (see Figure 1b in main text) than that in the Ca^{2+}-bound biosensor (Figure 1c), while 800 nm R_{pu} can achieve two-photon absorption of the protein biosensor chromophore [1,2].

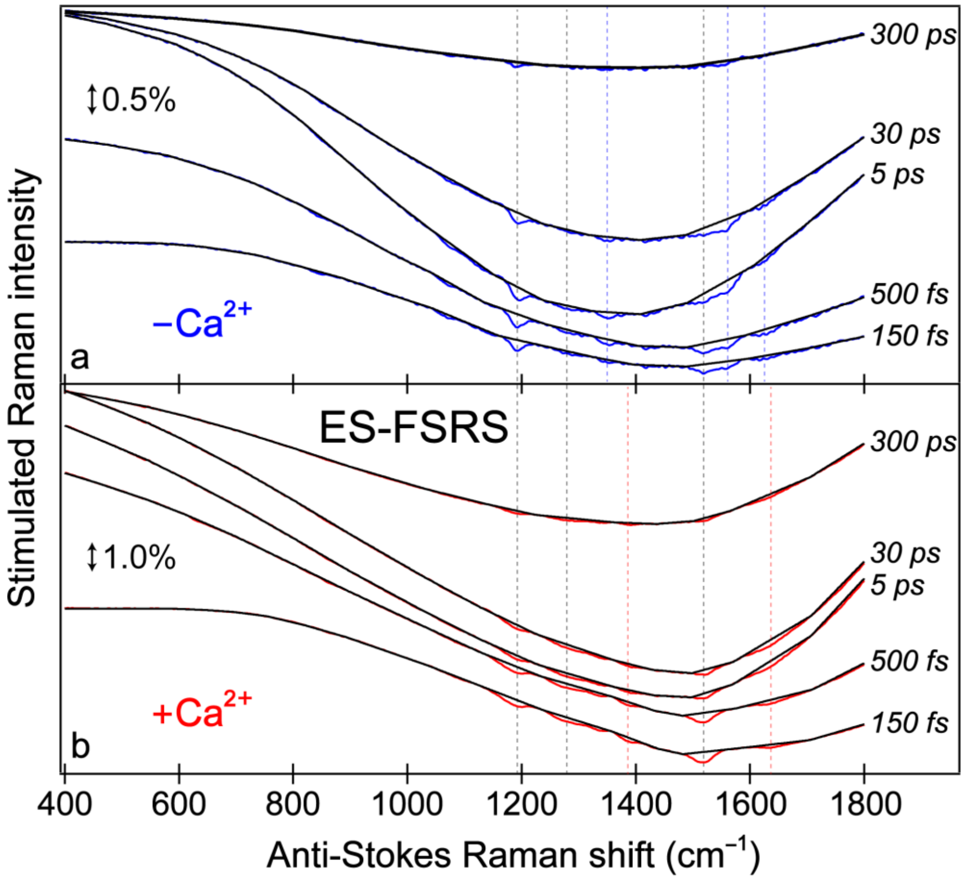

Figure S4. Excited-state FSRS spectra of the REX-GECO1 biosensor **(a)** without (blue, $-Ca^{2+}$) and **(b)** with (red, $+Ca^{2+}$) calcium ions in pH 7 buffer. Several representative time points at 150 fs, 500 fs, 5 ps, 30 ps, and 300 ps (marked on the right side) were selected from the time-resolved FSRS data with actinic pump and Raman pump wavelengths of 480 and 650 nm, respectively, along with a bluer Raman probe (i.e., on the anti-Stokes side) [3-6]. The stimulated Raman peaks (see blue and red traces, highlighted by vertical dashed lines) are shown on top of a broad underlying spectral baseline (black trace) that can be consistently subtracted at each time point. Besides vertical black dashed lines marking the conserved biosensor chromophore peaks regardless of Ca^{2+} binding, blue and red dashed lines denote prominent Raman peaks associated with the Ca^{2+}-free and Ca^{2+}-bound biosensors, respectively. The double-arrowed lines indicate the stimulated Raman intensity magnitude of 0.5% and 1.0% in panels **a** and **b**, respectively.

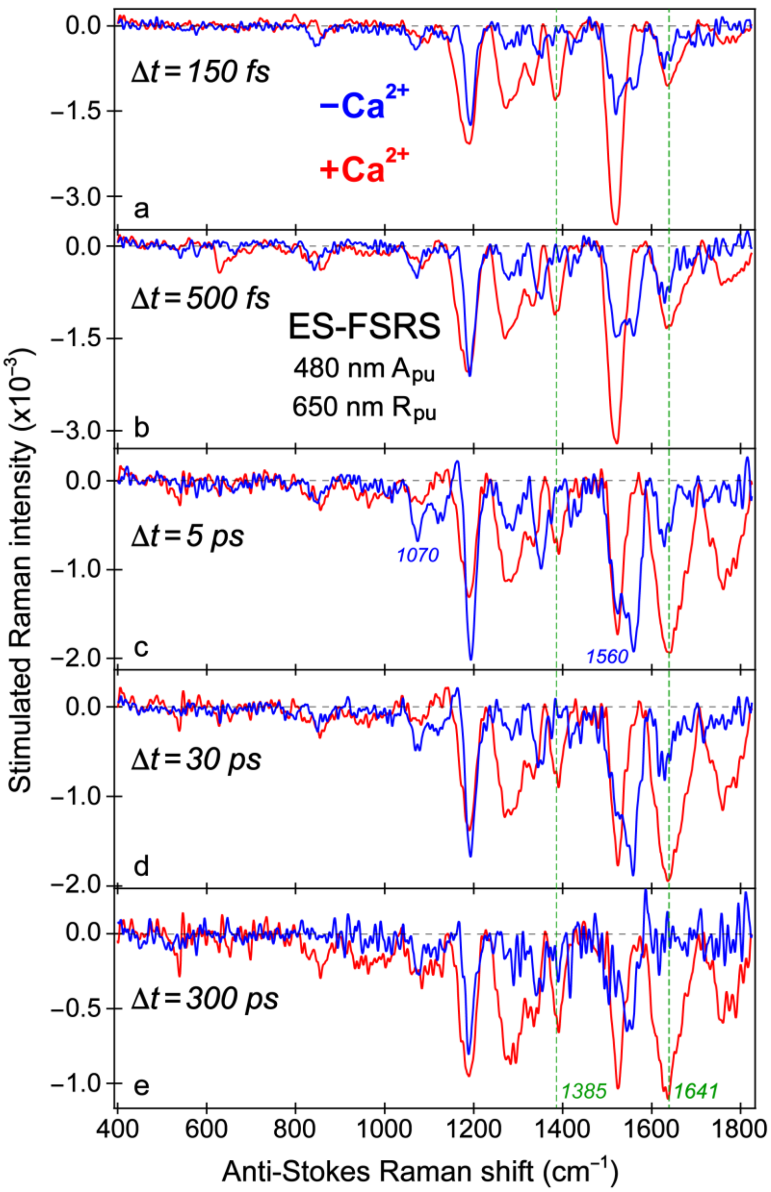

Figure S5. Selected traces from the baseline-subtracted excited-state FSRS spectra of the Ca^{2+}-free (blue) and Ca^{2+}-bound (red) REX-GECO1 biosensors in pH 7 buffer with a 480 nm actinic pump (A_{pu}) and 650 nm Raman pump (R_{pu}). The anti-Stokes Raman shift axis was multiplied by –1 to display positive frequency values. The selected time delays include **(a)** 150 fs, **(b)** 500 fs, **(c)** 5 ps, **(d)** 30 ps, and **(e)** 300 ps. The horizontal dashed lines indicate zero intensity, while the vertical thicker green dashed line tracks an ultrafast evolution of the ~1641 cm^{-1} marker band for the Ca^{2+}-bound biosensor which is distinct from its vibrational counterpart in the Ca^{2+}-free biosensor.

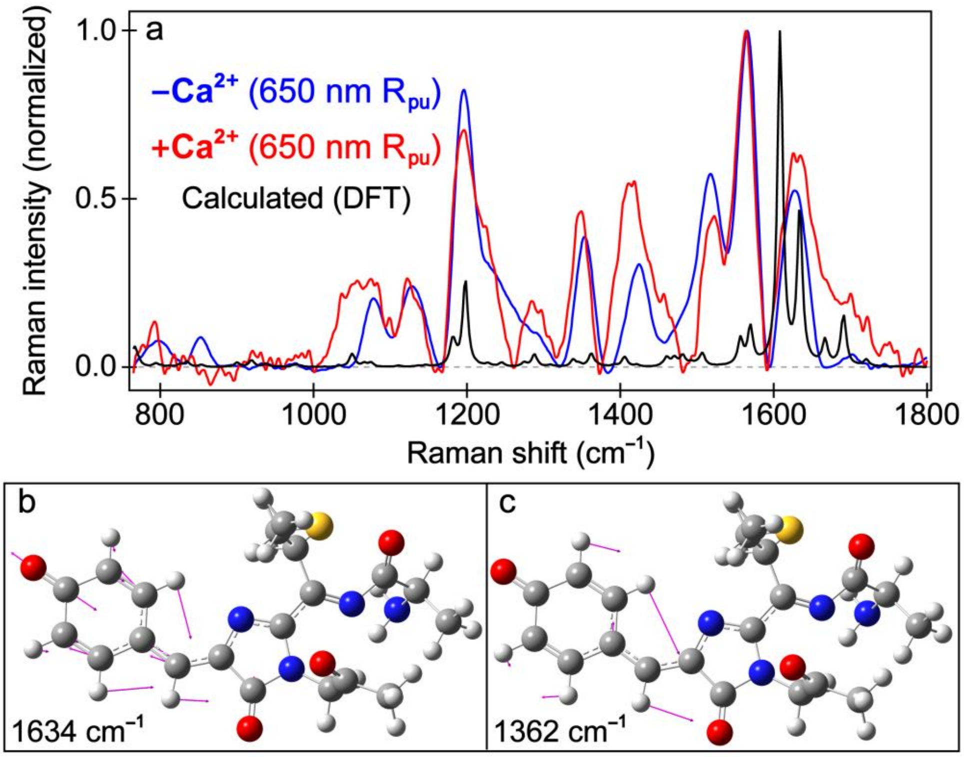

Figure S6. Calculated Raman and experimental ground-state FSRS spectra with depiction of key Raman modes. **(a)** Calculated Raman spectrum via density functional theory (DFT, frequency unscaled, black trace) of the deprotonated MYG chromophore *in vacuo* overlaid with the experimental Ca^{2+}-free (blue) and Ca^{2+}-bound (red) GS-FSRS spectra in pH 7 buffer with a 650 nm Raman pump (R_{pu}) on the anti-Stokes side (i.e., with a bluer Raman probe). The Raman shift axis of the experimental spectra was multiplied by –1 to display positive frequencies and compare to the calculated spectrum. All the spectra are normalized to allow for a better comparison, while the gray dashed line denotes zero intensity. Depiction of the representative **(b)** 1634 and **(c)** 1362 cm^{-1} Raman modes from vibrational normal mode calculations of the deprotonated MYG chromophore. The acylimine chain (with the –C=N–C=O moiety) at the imidazolinone (I)-ring end is explicitly shown and included in the calculations (see results tabulated in Tables S1 and S2 below). Atomic displacements from these key vibrational motions are depicted by magenta arrows.

2. Supplementary Tables

Table S1. Calculated ground state Raman with experimental ground and excited state FSRS peak frequencies of the Ca^{2+}-bound REX-GECO1 biosensor with vibrational normal mode assignments of the deprotonated methionine-tyrosine-glycine (MYG) chromophore *in vacuo*.

Calc. Freq. [a]	GS Exp. Freq. [b]	ES Exp. Freq. [c]	Mode Assignment (Major) [d]
1721/1712	1714	1771	I-ring C=O str.
1691/1682	1677	1683	Acylimine C=O str.
1634/1626	1631	1641	P-ring C=O and C=C str., bridge C=C str.
1570/1562	1562	–	Acylimine C=N str. with I-ring C=N str.
1557/1549	1521	1519	P-ring C=C str.
1460/1453	1452	–	Acylimine and I-ring sidechain H-sc.
1405/1398	1413	1385	H-motions throughout chromophore
1362/1355	1347	1331	P-ring H-rocking and H-sc. with bridge H-rocking
1288/1281	1288	1281	P-ring H-sc. and H-rocking, P- and I-ring def.
1227/1221	1228	–	Acylimine H-motions
1198/1192	1195	1184	P-ring H-sc. and bridge H-rocking
1136/1130	1127	–	Acylimine C–N stretch and H-motions
1075/1070	1078	1084	H-motions throughout chromophore, I-ring C–N str.
1050/1045	1045	–	Acylimine and I-ring sidechain H-motions
854/850	–	862	P-ring breathing
794/790	789	–	P-ring HOOP, H-motions throughout chromophore

[a] The unscaled/scaled (by a factor of 0.995) ground state (GS) Raman peak frequencies (in cm^{-1}) were calculated using the density functional theory (DFT)/B3LYP/6-31+G(d,p) as the method/functional/basis sets of the geometrically optimized chromophore structure. To focus on major peak assignments with an economical method using Gaussian software [7], we calculated the isolated chromophore in deprotonated state for comparison with the pre-resonantly enhanced deprotonated chromophore in the Ca^{2+}-bound REX-GECO1 biosensor (see Figure S1a), with the chromophore structure illustrated in Figure 1a (main text) [8].

[b] The GS-FSRS peak frequencies (in cm^{-1}) were determined by least-squares fitting the experimental spectra in pH 7 buffer solution with multiple gaussian profiles so the peak center frequencies can be extracted [9,10]. These peak frequencies can be directly viewed in Figure 5b (see red labels by the best-fit spectrum in main text) for the Ca^{2+}-bound biosensor.

[c] The ES-FSRS peak frequencies (in cm^{-1}) were determined by least-squares fitting the experimental spectra in pH 7 buffer solution with multiple gaussian profiles so the peak center frequencies at 300 fs time delay can be extracted. The 2D-contour plot for the Ca^{2+}-bound biosensor's ES-FSRS data is shown in Figure 6b (main text), and weak or absent peaks in either GS- or ES-FSRS spectra are denoted by "–" in this table.

[d] The abbreviations for key vibrational motions are: stretching (str.), scissoring (sc.), ring deformation (ring def.), hydrogen motions (H-motions), hydrogen rocking (H-rocking), and hydrogen-out-of-plane (HOOP) motion. Note that blue shading in this table highlights key ES-FSRS modes in the Ca^{2+}-bound biosensor.

Table S2. Calculated ground state Raman with experimental ground and excited state FSRS peak frequencies of the Ca^{2+}-free biosensor with vibrational normal mode assignments of the deprotonated MYG chromophore *in vacuo*.

Calc. Freq. [a]	GS Exp. Freq. [b]	ES Exp. Freq. [c]	Mode Assignment (Major) [d]
1721/1712	1701	–	I-ring C=O str.
1691/1682	–	–	Acylimine C=O str.
1634/1626	1628	1629	P-ring C=O and C=C str., bridge C=C str.
1570/1562	1565	1563	Acylimine C=N str. with I-ring C=N str.
1557/1549	1516	1524	P-ring C=C str.
1460/1453	1473	–	Acylimine and I-ring sidechain H-sc.
1405/1398	1425	1422	H-motions throughout chromophore
1362/1355	1353	1349	P-ring H-rocking and H-sc. with bridge H-rocking
1288/1281	1278	1281	P-ring H-sc. and H-rocking, P- and I-ring def.
1227/1221	1233	–	Acylimine H-motions
1198/1192	1196	1193	P-ring H-sc. and bridge H-rocking
1136/1130	1128	–	Acylimine C–N stretch and H-motions
1075/1070	1078	1070	H-motions throughout chromophore, I-ring C–N str.
1050/1045	–	–	Acylimine and I-ring sidechain H-motions
854/850	853	847	P-ring breathing
794/790	797	–	P-ring HOOP, H-motions throughout chromophore

[a] The unscaled/scaled (by a factor of 0.995) ground state (GS) Raman peak frequencies (in cm^{-1}) were calculated using the DFT/B3LYP/6-31+G(d,p) as the method/functional/basis sets of the geometrically optimized chromophore structure in Gaussian. We calculated the isolated, deprotonated MYG chromophore for comparison with the pre-resonantly enhanced deprotonated chromophore in the Ca^{2+}-free REX-GECO1 biosensor (see Figure S1a), with the chromophore structure illustrated in Figure 1a (main text).

[b] The GS-FSRS peak frequencies (in cm^{-1}) were determined by least-squares fitting the experimental spectra in pH 7 buffer with multiple gaussian profiles so the peak center frequencies can be extracted. These peak frequencies can be viewed in Figure 5a (see blue labels by the best-fit spectrum in main text) for the Ca^{2+}-free biosensor, its peak intensities dominating the Ca^{2+}-bound counterpart due to resonance conditions.

[c] The ES-FSRS peak frequencies (in cm^{-1}) were determined by least-squares fitting the experimental spectra in pH 7 buffer solution with multiple gaussian profiles so the peak center frequencies at 300 fs time delay can be extracted. The 2D-contour plot for the Ca^{2+}-free biosensor's ES-FSRS data is shown in Figure 6a (main text), and weak or absent peaks in either GS- or ES-FSRS spectra are denoted by "–" in this table.

[d] The abbreviations for key vibrational motions are: stretching (str.), scissoring (sc.), ring deformation (ring def.), hydrogen motions (H-motions), hydrogen rocking (H-rocking), and hydrogen-out-of-plane (HOOP) motion. Note that blue shading in this table highlights the important ES-FSRS marker bands in the Ca^{2+}-free biosensor, also marked by vertical dashed lines in Figure 6a (see main text).

3. Supplementary References

1. Drobizhev, M.; Makarov, N.S.; Tillo, S.E.; Hughes, T.E.; Rebane, A. Two-photon absorption properties of fluorescent proteins. *Nat. Meth.* **2011**, *8*, 393–399.
2. Tang, L.; Liu, W.; Wang, Y.; Zhu, L.; Han, F.; Fang, C. Ultrafast structural evolution and chromophore inhomogeneity inside a green-fluorescent-protein-based Ca^{2+} biosensor. *J. Phys. Chem. Lett.* **2016**, *7*, 1225–1230.
3. Dietze, D.R.; Mathies, R.A. Femtosecond stimulated Raman spectroscopy. *ChemPhysChem* **2016**, *17*, 1224–1251.
4. Batignani, G.; Pontecorvo, E.; Giovannetti, G.; Ferrante, C.; Fumero, G.; Scopigno, T. Electronic resonances in broadband stimulated Raman spectroscopy. *Sci. Rep.* **2016**, *6*, 18445.
5. Liu, W.; Tang, L.; Oscar, B.G.; Wang, Y.; Chen, C.; Fang, C. Tracking ultrafast vibrational cooling during excited state proton transfer reaction with anti-Stokes and Stokes femtosecond stimulated Raman spectroscopy. *J. Phys. Chem. Lett.* **2017**, *8*, 997–1003.
6. Tang, L.; Zhu, L.; Taylor, M.A.; Wang, Y.; Remington, S.J.; Fang, C. Excited state structural evolution of a GFP single-site mutant tracked by tunable femtosecond-stimulated Raman spectroscopy. *Molecules* **2018**, *23*, 2226.
7. Frisch, M.J.; Trucks, G.W.; Schlegel, H.B.; Scuseria, G.E.; Robb, M.A.; Cheeseman, J.R.; Scalmani, G.; Barone, V.; Petersson, G.A.; Nakatsuji, H.; et al. *Gaussian 16, Revision A.03*; Gaussian, Inc.: Wallingford, CT, USA, 2016.
8. Akerboom, J.; Calderon, N.C.; Tian, L.; Wabnig, S.; Prigge, M.; Tolo, J.; Gordus, A.; Orger, M.B.; Severi, K.E.; Macklin, J.J.; et al. Genetically encoded calcium indicators for multi-color neural activity imaging and combination with optogenetics. *Front. Mol. Neurosci.* **2013**, *6*, 2.
9. Fang, C.; Tang, L.; Oscar, B.G.; Chen, C. Capturing structural snapshots during photochemical reactions with ultrafast Raman spectroscopy: From materials transformation to biosensor responses. *J. Phys. Chem. Lett.* **2018**, *9*, 3253–3263.
10. Fang, C.; Tang, L. Mapping structural dynamics of proteins with femtosecond stimulated Raman spectroscopy. *Annu. Rev. Phys. Chem.* **2020**, *71*, 239–265.
